# Accurate Thermochemical and Kinetic Parameters at Affordable Cost by Means of the Pisa Composite Scheme (PCS)

**DOI:** 10.1021/acs.jctc.3c00817

**Published:** 2023-09-29

**Authors:** Vincenzo Barone, Luigi Crisci, Silvia Di Grande

**Affiliations:** † Scuola Normale Superiore di Pisa, Piazza dei Cavalieri 7, 56125 Pisa, Italy; ‡ Scuola Superiore Meridionale, Largo San Marcellino 10, 80138 Napoli, Italy

## Abstract

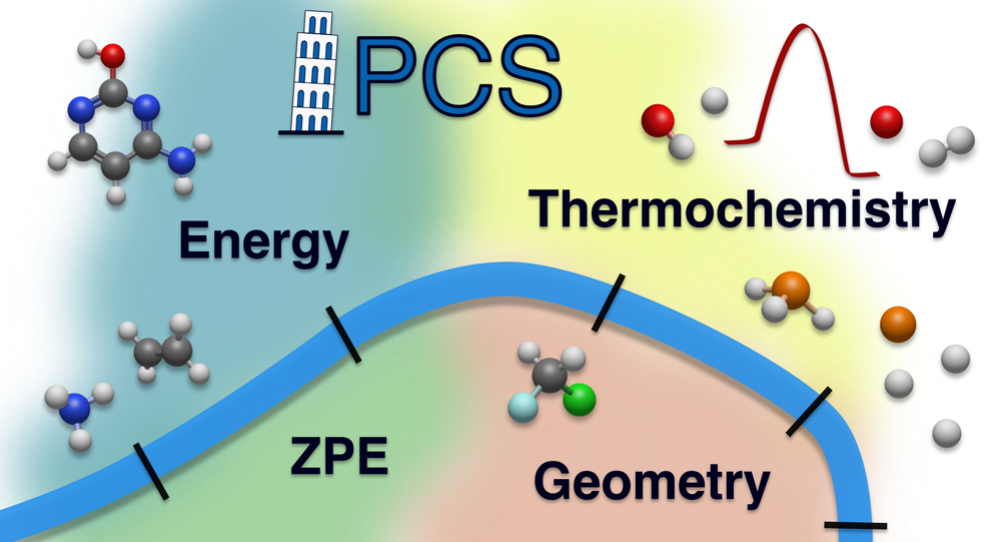

A new strategy for the computation at an affordable cost of geometrical structures, thermochemical parameters, and rate constants for medium-sized molecules in the gas phase is proposed. The most distinctive features of the new model are the systematic use of cc-pV*n*Z-F12 basis sets, the addition of MP2 core–valence correlation in geometry optimizations by a double-hybrid functional, the separate extrapolation of MP2 and post-MP2 contributions, and the inclusion of anharmonic contributions in zero-point energies and thermodynamic functions. A thorough benchmark based on a wide range of prototypical systems shows that the new scheme outperforms the most well-known model chemistries without the need for any empirical parameter. Additional tests show that the computed zero-point energies and thermal contributions can be confidently used for obtaining accurate thermochemical and kinetic parameters. Since the whole computational workflow is translated in a black-box procedure, which can be followed with standard electronic structure codes, the way is paved for the accurate yet not prohibitively expensive study of medium- to large-sized molecules also by nonspecialists.

## 1 Introduction

The main factors determining the accuracy of computed thermochemical and kinetic parameters are the reaction energies and the energy barriers for all of the elementary steps involved in the process under investigation. In the absence of species with strong multireference character and/or nonadiabatic effects, the coupled cluster (CC) approach^1^ delivers accurate results provided that the most important classes of excitations are included together with complete basis set (CBS) extrapolation, core–valence (CV) correlation and, if needed, other minor effects (scalar relativistic, diagonal Born–Oppenheimer, spin–orbit). Due to an effective error compensation, single, double, and perturbative estimates of triple excitations are usually sufficient,^2^ leading to the CCSD(T)-CBS+CV model, which is often considered the gold standard of contemporary computational chemistry. At this level, chemical accuracy (4 kJ mol^–1^) can be reached by employing large basis sets (e.g., FPA^3,4^), resorting to empirical parameters in conjunction with smaller basis sets (e.g., G4,^5^ CBS-QB3^6^), or employing explicitly correlated (F12) models (e.g., W1–F12^7^ or SVECV-F12^8^). The most reliable protocols (e.g., HEAT,^9^ W4,^10^ and their explicitly correlated HEAT-F12^11^ and W4–F12^12^ variants) further increase the overall accuracy (below 1 kJ mol^–1^) including additional (expensive) contributions. In this connection, it should be pointed out that the latter protocols also push geometry optimizations to the limit, whereas, at the other extreme, G4 and CBS-QB3 schemes employ B3LYP geometries, whose accuracy is often unsatisfactory.^5,13^

Next, zero-point energies (ZPEs) and finite temperature contributions (FTCs) come into play, which are determined by the geometries and vibrational frequencies. In this connection, effective approaches going beyond the standard rigid-rotor/harmonic-oscillator (RRHO) model are needed, especially when light atoms or hindered rotors are involved. Semirigid systems are well described by second-order vibrational perturbation theory (VPT2)^14−16^ and weakly coupled large-amplitude motions can be included by effective one-dimensional models.^17^ Finally, the noncovalent interactions playing a key role in the formation of weak complexes can be treated by classical stochastic models.^18^ More challenging situations requiring more refined models will not be considered in the present paper.

Based on these premises, we have developed a composite method, referred to as the Pisa composite scheme (PCS), devoid of any empirical parameter (besides those possibly present in the underlying electronic structure method), which should provide accurate structural and energetic data at nonprohibitive costs. In analogy with the W1X^19^ and SVECV-F12^8^ composite methods, PCS employs second-order Møller–Plesset perturbation theory (MP2)^20^ for estimating CV correlation. Further reduction of the computational cost is achieved by performing separate CBS extrapolations for MP2 and post-MP2 contributions.

In the present paper, we describe the essential details of the PCS model and perform a comprehensive benchmark of its performance for several systems for which accurate reference results are available or have been purposely computed. We show that this model chemistry offers a remarkable compromise between accuracy and feasibility for medium- to large-sized molecules. Together with electronic energies, we also analyze the role of geometries, ZPEs, and FTCs in tuning thermochemical and kinetic parameters.

## 2 Methods

### 2.1 PCS Model

As mentioned in Section 1, starting from CCSD(T) computations in conjunction with a sufficiently large basis set, CBS extrapolation can be performed by resorting either to lower-level models (typically MP2) in the spirit of the focal point approach (FPA)^4^ or to explicitly correlated (F12) models.^21^ In the present study, we will show that the first route can still be competitive with the second one, which has been recently privileged. To this end, we resort to basis sets explicitly optimized for F12 computations, namely, the cc-pV*n*Z-F12 family^22^ (hereafter *n*F12), which has been recently shown^23−25^ to approach the accuracy of augmented (*n*+1)-zeta conventional basis sets with a reduced cost also for DFT and conventional post-Hartree–Fock models. Furthermore, on the grounds of previous experience,^13,26^ the CBS extrapolation of HF and MP2 contributions is performed in a single step. Therefore, the starting point of the new PCS model is a frozen core (fc) CCSD(T) computation in conjunction with the 3F12 basis set. Next, CV correlation is computed at the MP2 level in conjunction with the cc-pwCVTZ basis set^27,28^ (hereafter wC3) and the CBS extrapolation is performed employing the 3F12 and 4F12 basis sets for the MP2 contribution, whereas the 2F12 and 3F12 basis sets are employed for the difference between CCSD(T) and MP2 energies. Both CBS extrapolations are performed by the standard *n*^–3^ two-point formula.^29^ After the rearrangement of some terms, the complete PCS energy can be conveniently written in the following way:
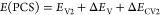
1where
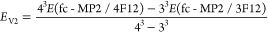
2
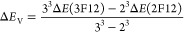
3with
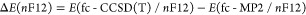
4and
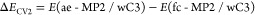
5where ae stands for all electrons. Finally, the experimental values of spin–orbit couplings are employed for O, OH, SH, and Cl radicals, lowering their electronic energies by 0.9, 0.8, 2.3, and 3.5 kJ mol^–1^, respectively.^30^

Benchmark computations have been performed by an explicitly correlated version of the method (PCS-F12),^31^ which is obtained by replacing conventional MP2 and CCSD(T) steps with their MP2-F12^32^ and CCSD(T)-F12^33,34^ counterparts in the *E*_*V*2_ and Δ*E*_*V*_ terms. In particular, the CCSD(F12*) model of Hattig and co-workers was employed^35^ in conjunction with the size-consistent (T+) correction for the triple excitations.^34^ For comparison purposes, the simpler F12b model has been used in conjunction with the standard perturbative inclusion of triple excitations.^33,36^ The cc-pV*n*Z-F12-OPTRI CABS were employed for resolution of identity (RI) and inclusion of the contributions of the complementary auxiliary basis set (CABS).^37,38^ The density fitting (DF) approximation was used throughout the HF and the correlation calculations employing the aug-cc-pV(*n*+1)Z-RI-JK^39^ and aug-cc-pwCV(*n*+1)Z-RI^40^ fitting basis sets, respectively. Slater-type *f*_12_ correlation factors (fitted with 6 Gaussians each) with exponents of 0.9, 1.0, and 1.1 were employed for the 2F12, 3F12, and 4F12 basis sets, respectively.^41^

In the case of open-shell systems the spin contamination from higher-spin excited states can be so large to strongly distort potential energy surfaces computed by unrestricted wave functions,^42^ making problematic the use of CBS and CV contributions evaluated at the UMP2 level. While the *S*+1 contaminant is fully removed at the UCCSD level^43^ and the *S*+2 contaminant is significantly reduced at the UCCSD(T) level,^44^ in extreme cases, also the UCCSD(T) results can become unreliable.^45^ As a consequence, all of the steps of the PCS approach are performed employing the restricted open-shell formalism.

Derivation of eq 1 w.r.t. Cartesian coordinates leads to PCS energy gradients, which can be employed for geometry optimizations by composite methods.^46,47^ However, a much simpler approach (referred to as geometry scheme^48,49^) is obtained assuming that the additivity approximation can be directly applied to geometrical parameters (*r*) and only involves geometry optimizations at several levels of theory. The geometry scheme does not require any modification of standard electronic structure codes, is embarrassingly parallel, and, according to a detailed benchmark,^50^ provides results nearly identical to those delivered by the gradient scheme. A cheaper alternative (PCS/DFT) is obtained by replacing the CCSD(T)/3F12 contribution with its revDSD-PBEP86-D3(BJ)/3F12 counterpart^51−53^ (this combination of functional and basis set will be referred to in the following simply as rDSD). In this case, recent work has shown that CBS extrapolation can be neglected^24^ so that
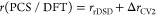
6with
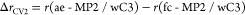
7This version is particularly effective since low-scaling methods or analytical implementations are widely available for MP2 (or double-hybrid density functionals) gradients and several recent studies have confirmed that PCS/DFT geometries are sufficiently accurate for most applications.^24,31,54^ Concerning open-shell systems, it has been shown that the opposite behavior of UHF and UMP2 contributions with respect to spin contamination leads to very stable results when using double-hybrid functionals.^55^ As a consequence, rDSD gradients and Hessians have always been computed by employing the unrestricted formalism.

In general terms, the PCS and PCS/DFT models can be regarded as improved versions of the already highly successful junChS model,^13,26^ which is recovered when the *n*F12 basis sets are replaced by their jun-cc-pV*n*Z^56^ counterparts (hereafter j*n*), CV correlation is neglected in geometry optimizations, and Δ*E*_V_ = Δ*E*(j3).

ZPEs have also been computed (in order to get enthalpies at 0 K) employing rDSD harmonic and anharmonic contributions in the framework of vibrational perturbation theory to second order (VPT2).^57^ The same terms have been employed to compute partition functions (hence thermal contributions to thermochemical and kinetic parameters) in the framework of the so-called simple perturbation theory (SPT)^58^ in conjunction with one-dimensional models for the treatment of hindered rotors.^59^ Further details are given in the following sections.

All of the conventional computations have been performed with the Gaussian package,^60^ whereas explicitly correlated computations and evaluation of additional terms (see next section), have been performed with the MOLPRO^61^ and MRCC^62^ codes.

### 2.2 Additional Terms

Starting from PCS electronic energies, additional terms can be added to obtain high-accuracy (*H*) results (*E*_HPCS_)
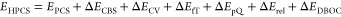
8

The CBS and CV contributions refer to the differences between the evaluations of these terms at the CCSD(T) and MP2 levels. The diagonal Born–Oppenheimer correction Δ*E*_DBOC_^63−66^ and the scalar relativistic contribution to the energy Δ*E*_rel_^67,68^ are computed at the HF-SCF/aug-cc-pVDZ^69^ and CCSD(T)/aug-cc-pCVDZ^27^ level. Finally, the corrections due to full treatment of triple (Δ*E*_fT_) and perturbative treatment of quadruple (Δ*E*_pQ_) excitations are computed, within the fc approximation, as energy differences between CCSDT and CCSD(T) and between CCSDT(Q) and CCSDT calculations employing the cc-pVTZ and cc-pVDZ basis set,^70^ respectively.

While straightforward generalizations of eq 6 would allow geometry optimizations at the HPCS level, this route has not been pursued here due to the negligible improvement over PCS geometries in most cases and the cost (fT) or lack (pQ) of analytical gradients.

## 3 Results and Discussion

In the next subsection, the performances of the PCS model for thermochemistry will be analyzed by employing the test set of Knizia, Adler, and Werner (KAW).^33^ Next, reaction barriers will be investigated, followed by inter- and intramolecular noncovalent interactions, together with the stability of different tautomeric forms. Then, after benchmarks of geometrical parameters and harmonic frequencies were analyzed, zero-point energies, thermodynamic functions, and reaction rate constants are analyzed.

### 3.1 Thermochemistry

The first analysis of the PCS performance refers to the KAW test set, which includes 49 atomization energies together with 28 closed-shell and 48 open-shell reaction energies. Since the reference values of the KAW test set do not contain the CV correlation, the CV2 term is not considered in this case. The errors delivered by PCS are compared in Table 1 with those issued from the underlying CCSD(T)/3F12 computations and from other models in terms of maximum difference (MAX), mean unsigned error (MUE) and root-mean-square deviation (RMSD). The complete results are given in Tables S1 and S4 in the Supporting Information (SI).

**Table 1:** Maximum Difference (MAX), Mean Unsigned Error (MUE), and Root-Mean-Square Deviation (RMSD) for the KAW Test set with Respect to the CCSD(T)/aug-cc-pV(5,6)Z Results of Ref (33)^a^

	CC/3F12	PCS	CC/J3	junChS	CC-F12/3F12	PCS-F12	CC-F12/J3^b^	junChS-F12^b^
atomization energies								
MAX	53.7	8.8	70.0	10.6	9.9	8.4	12.9 (16.1)	10.3 (13.9)
MUE	25.0	2.9	30.7	4.0	3.7	2.3	4.2 (6.6)	2.7 (5.3)
RMSD	27.2	3.6	33.5	4.8	4.5	3.4	5.1 (7.4)	3.8 (6.0)
closed-shell react. energies								
MAX	35.7	7.6	30.1	5.2	6.0	3.6	5.0 (6.2)	5.3 (3.4)
MUE	7.3	1.6	5.4	1.5	1.0	0.8	1.4 (1.2)	1.1 (0.8)
RMSD	10.0	2.2	8.3	2.1	1.6	1.2	2.0 (1.7)	1.7 (1.2)
open-shell react. energies								
MAX	50.5	6.3	70.1	11.6	7.2	6.3	6.4 (7.8)	6.6 (13.7)
MUE	14.6	1.9	18.5	3.0	1.8	1.7	2.0 (3.1)	2.1 (2.8)
RMSD	18.0	2.5	22.5	4.4	2.5	2.4	2.6 (3.8)	2.8 (3.7)
total								
MAX	53.7	8.8	70.1	11.6	9.9	8.4	12.9 (16.1)	10.3 (13.9)
MUE	17.1	2.2	20.3	3.1	2.4	1.7	2.7 (4.0)	2.1 (3.3)
RMSD	20.9	2.9	25.5	4.0	3.3	2.6	3.7 (5.5)	3.1 (4.5)

a CC stands for CCSD(T) and CC-F12 for CCSD(F12*)(T+). All of the numerical values are in kJ mol^–1^.

b CCSD(F12*)(T+) and, in parentheses, CCSD(T)-F12b from ref (71).

As is well known, atomization energies represent the most challenging benchmarks, and actually, all of the conventional CCSD(T) approaches deliver quite disappointing results, with total MUEs around 20 kJ mol^–1^. Then, the CBS extrapolation of both MP2 and post-MP2 contributions reduces the error by nearly an order of magnitude, reaching the so-called chemical accuracy (MUE of 4 kJ mol^–1^).

This result is indeed remarkable in view of the negligible cost of the extrapolations with respect to the underlying coupled clusters computations. The general trends are the same for both closed- and open-shell reaction energies, with this behavior being particularly relevant for the robustness and generality of the computational approach. The improvement of PCS with respect to junChS is quite apparent: for instance, the MUE is reduced by about 30% (from 3.1 to 2.2 kJ mol^–1^) and also the corresponding RMSD (2.9 kJ mol^–1^) now falls well below the so-called chemical accuracy.

The effectiveness of both the 3F12 basis set and the MP2 extrapolation is further confirmed by the comparison to explicitly correlated computations. As a matter of fact, the error statistics of PCS and PCS-F12 or junChS and junChS-F12 computations are quite similar, whereas this is not the case for the starting coupled cluster computations. Noted is that the accuracy of different approximate models for the CCSD-F12 step and, especially, the perturbative inclusion of triple contributions can lead to non-negligible differences (see Table 1). As a matter of fact, only the accurate and size-extensive CCSD(F12*)(T+) variant offers a slight improvement over the corresponding conventional approach when including CBS extrapolation, which, as already mentioned, is much more important for conventional (1 order of magnitude reduction of the error) than for explicitly correlated (40% reduction of the error) methods but is not negligible also in the latter case. Furthermore, the contribution of the 3F12/4F12 extrapolation of the MP2 (or MP2-F12) energy is about half of the 2F12/3F12 extrapolation of the post-MP2 terms (see Table S4 in the SI). In conclusion, both the MP2 and F12 routes to CBS extrapolation can be followed with comparable cost and performance.

### 3.2 Reaction Barriers

The most widely employed reference results for reaction barriers are collected in the DBH24 compilation^72,73^ containing results mostly obtained at the CCSDTQ5/CBS level by means of the W4 composite method^10^ for a statistically representative set including 3 prototypes for each of the following classes of reactions: heavy-atom transfer, nucleophilic substitution, unimolecular and association reactions, and hydrogen-transfer reactions.

Table 2 compares the reaction barriers computed at the PCS level to the reference values of ref (73). In this case, the CCSD(T)/3F12 error is already quite small and is further reduced by adding CBS and CV contributions with inexpensive computations. These trends confirm that two-point extrapolations are effective routes for estimating the CBS limit without introducing additional computational bottlenecks with respect to the underlying CCSD(T)/3F12 reference. The average contribution of triple and quadruple excitations (about 1 kJ mol^–1^) defines a lower error boundary for methods (like PCS), not including these contributions. Actually, the PCS MUE (1.2 kJ mol^–1^) is lower than the corresponding junChS value (1.5 kJ mol^–1^) and close to the expected lower boundary with most of the improvement being related to the replacement of CCSD(T)/j3 with CCSD(T)/3F12. The W1X method, which has computational costs comparable to those of PCS, delivers a similar MUE (1.3 kJ mol^–1^),^74^ and the same applies to the explicitly correlated SVECV-F12 model^8^ (MUE of 1.1 kJ mol^–1^). Although other explicitly correlated approaches further reduce the MUE (1.0, 0.9, and 0.8 kJ mol^–1^ at the junChS-F12,^75^ W1–F12^74^ and PCS-F12 level, respectively) this further improvement is not significant in view of the error associated with the neglect of full-triple and quadruple excitations. Contrary to atomization energies, the results are not particularly sensitive to the approximations employed in the CCSD(T)-F12 ansatz and to the accuracy of the underlying optimized geometries. Notably, the anharmonic contributions to ZPE differences (together with spin–orbit contributions) are sometimes comparable to those of their (fT+pQ) counterparts.

**Table 2:** Theoretical Values of the Barrier Heights (Including Spin–Orbit Correction and not ZPEs) in the DBH24 Compilation Obtained at Different Levels of Theory^a^

	reactions	forward/reverse barrier height						
		junChS^b^^,^^c^	PCS^*a*^	CC/j3^b^^,^^c^	CC/3F12^b^	fT+pQ	ref (73).	Δanh
heavy-atom transfer								
a1^d^	H^•^ + N_2_O → OH^•^ + N_2_	73.3/348.3	72.6/347.7	74.9/355.5	74.7/353.7	–1.4/–3.2	71.7/345.0	–0.2/–0.2
a2	H^•^ + ClH → HCl + H^•^	72.4/72.4	72.5/72.5	79.0/79.0	77.4/77.4	–0.5/–0.5	75.3/75.3	1.3/1.3
a3^d^	CH_3_^•^ + FCl → CH_3_F + Cl^•^	30.0/252.6	29.8/253.7	30.2/260.2	29.9/255.8	–1.0/–1.2	28.2/251.0	–0.8/0.1
nucleophilic substitution								
a4	Cl^–^···CH_3_Cl → ClCH_3_···Cl^–^	55.5/55.5	56.6/56.6	56.7/56.7	54.9/54.9	–1.0/–1.0	56.1/56.1	0.1/0.1
a5	F^–^···CH_3_Cl → FCH_3_···Cl^–^	14.2/121.7	14.5/122.5	14.7/123.3	14.1/120.2	–0.8/–0.9	14.4/123.1	0.0/0.0
a6	OH^–^ + CH_3_F → HOCH_3_+ F^–^	–10.4/72.6	–12.0/72.6	–10.0/74.4	–12.2/71.2	–1.3/–1.4	–10.2/73.9	–0.4/0.0
unimolecular and association								
a7	H^•^ + N_2_ → HN_2_^•^	60.0/46.5	59.8/46.2	63.7/46.1	63.2/43.7	0.5/0.7	60.1/44.4	–0.4/0.1
a8	H^•^ + C_2_H_4_ → C_2_H_5_^•^	7.9/176.5	7.8/176.8	10.2/178.2	9.1/174.9	–0.5/–0.6	7.2/174.7	–0.3/–0.2
a9	HCN ↔ HNC	200.7/139.1	201.1/137.7	198.5/137.1	198.3/135.4	–0.2/–0.6	201.1/137.3	0.1/0.1
hydrogen transfer								
a10^e^	OH^•^ + CH_4_ → CH_3_^•^ + H_2_O	27.7/83.9	27.7/81.8	29.5/79.7	29.1/79.0	–0.7/–0.6	28.1/82.0	1.0/0.9
a11^d^^,^^e^	H^•^ + OH^•^ → H_2_ + ^3^O	48.2/57.6	45.7/56.4	43.4/61.2	43.4/60.9	–0.6/–1.0	44.8/54.9	0.7/0.8
a12^d^	H^•^ + H_2_S → H_2_ + HS^•^	15.5/75.1	15.5/75.2	17.7/80.5	15.9/76.7	–0.5/–0.4	15.2/72.5	–0.5/–0.6
MAX^f^		3.3/3.3 (3.3)	2.8/2.8 (2.8)	3.7/10.4 (10.4)	3.1/8.6 (8.6)			
MUE^f^		1.0/2.0 (1.5)	0.9/1.6 (1.2)	2.0/3.9 (3.0)	1.8/3.2 (2.5)			
RMSD^f^		1.5/2.1 (1.8)	1.2/1.9 (1.5)	2.4/5.3 (4.1)	2.0/3.9 (3.1)			

a The contributions of full-triple and perturbative-quadruple excitations (fT+pQ) and the differences between anharmonic and harmonic ΔZPEs computed at the rDSD/3F12 level (Δanh) are also given. CC stands for CCSD(T), and all of the numerical values are in kJ mol^–1^.

b At rDSD/j3 geometries.

c From ref (13).

d Spin–orbit contributions on the reverse reaction barrier.

e Spin–orbit contributions on the forward reaction barrier.

f Forward/reverse barrier height errors and (in parentheses) total values.

### 3.3 Noncovalent Interactions and Tautomeric Equilibria

After validating the PCS model for the thermochemistry and reaction barriers of small systems, we proceed with checking its performances for inter- and intramolecular noncovalent interactions.

**Figure 1:**
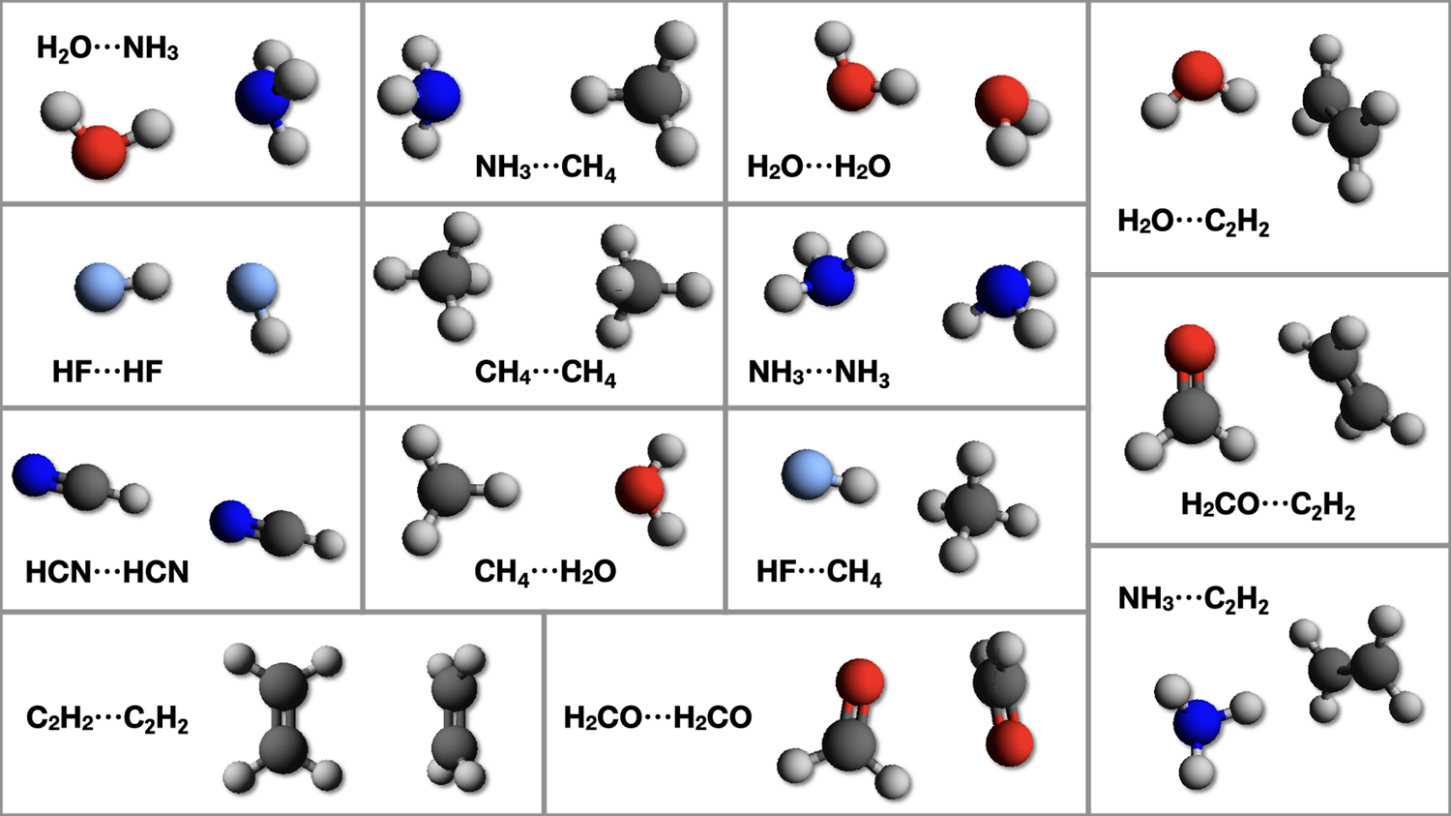
Noncovalent complexes of the A14 database.^77^

The noncovalent complexes belonging to the A14 set^26^ are shown in Figure 1, and the corresponding interaction energies obtained by different methods are collected in Table 3. The results show that the PCS model reaches an MUE of 0.16 kJ mol^–1^ without the need for any correction for the basis set superposition error (BSSE). Once again, a slight improvement is observed with respect to the junChS results (MUE of 0.22 kJ mol^–1^), with both PCS and junChS results confirming that the inclusion of CBS extrapolation and CV correlation increases the accuracy of the underlying coupled cluster model without any significant increase in computational requirements. Noted is that an average error of 0.2 kJ mol^–1^ is considered the gold standard for noncovalent interactions.^76^

**Table 3:** Interaction Energies of the A14 Complexes Obtained at Different Levels of Theory at the rDSD/j3 Geometries^a^

	reference^b^	junChS^b^	PCS	CC/j3^b^	CC/3F12
H_2_O···H_2_O	–21.08	–21.37	–21.22	–21.02	–21.32
NH_3_···NH_3_	–13.21	–13.34	–13.43	–12.78	–13.18
HF···HF	–19.22	–19.59	–19.59	–19.38	–19.53
HCN···HCN	–19.98	–19.70	–19.96	–20.54	–19.77
CH_4_···CH_4_	–2.23	–2.22	–2.27	–2.09	–2.21
CH_2_O···CH_2_O	–18.93	–19.43	–19.41	–18.27	–18.52
C_2_H_4_···C_2_H_4_	–4.60	–4.78	–4.76	–4.73	–4.62
H_2_O···C_2_H_4_	–10.77	–11.01	–10.77	–10.79	–11.02
H_2_O···CH_4_	–2.82	–2.79	–2.78	–2.86	–2.83
H_2_O···NH_3_	–27.38	–27.69	–27.59	–27.23	–27.51
NH_3_···CH_4_	–3.22	–3.24	–3.22	–3.28	–3.29
NH_3_···C_2_H_4_	–5.79	–5.99	–5.88	–5.81	–5.90
HF···CH_4_	–6.92	–7.14	–7.12	–7.07	–7.29
C_2_H_4_···CH_2_O	–6.79	–7.07	–7.05	–6.87	–6.68
MAX		0.50	0.48	0.66	0.41
MUE		0.22	0.16	0.19	0.16
RMSD		0.26	0.21	0.27	0.21
MAX%		4.10	3.68	6.28	5.36
MUE%		2.06	1.65	1.95	1.55

a CC stands for CCSD(T), and all of the values are in kJ mol^–1^.

b From ref (80).

Intramolecular noncovalent interactions are examined by analyzing the conformational landscape of the prototypical glycine amino acid (see Table 4). Several studies have shown that up to eight different energy minima are present in the glycine conformational potential energy surface (PES).^78^ Comparison with the very accurate results of ref (79). (roughly corresponding to HPCS values) shows that the PCS model performs a remarkable job, with MAX and MUE between the two models being 0.15 and 0.07 kJ mol^–1^, respectively. The quantitative agreement between HPCS and PCS results is not unexpected since conformational equilibria are already quite well described by low-level QC methods: as a matter of fact, already rDSD relative energies are usually sufficient for semiquantitative analyses of this kind of problems.^78^

**Table 4:** Relative Electronic Energies of the Eight Conformers of Glycine Computed by Different Methods^a^

conformer	reference^b^	PCS	rDSD/3F12
I (ttt)	0.0	0.0	0.0
II (ccc)	2.7	2.7	2.5
I′ (gtt)	5.2	5.1	5.3
III (tct)	7.2	7.4	7.0
III′ (gct)	11.1	11.2	11.0
Ic (ttc)	20.1	20.2	20.1
IIIc (tcc)	24.4	24.6	24.2
I′c (gtc)	25.4	25.4	25.5

a The conformer nomenclature is taken from ref (78), and all of the values are in kJ mol^–1^.

b From ref (79).

**Figure 2:**
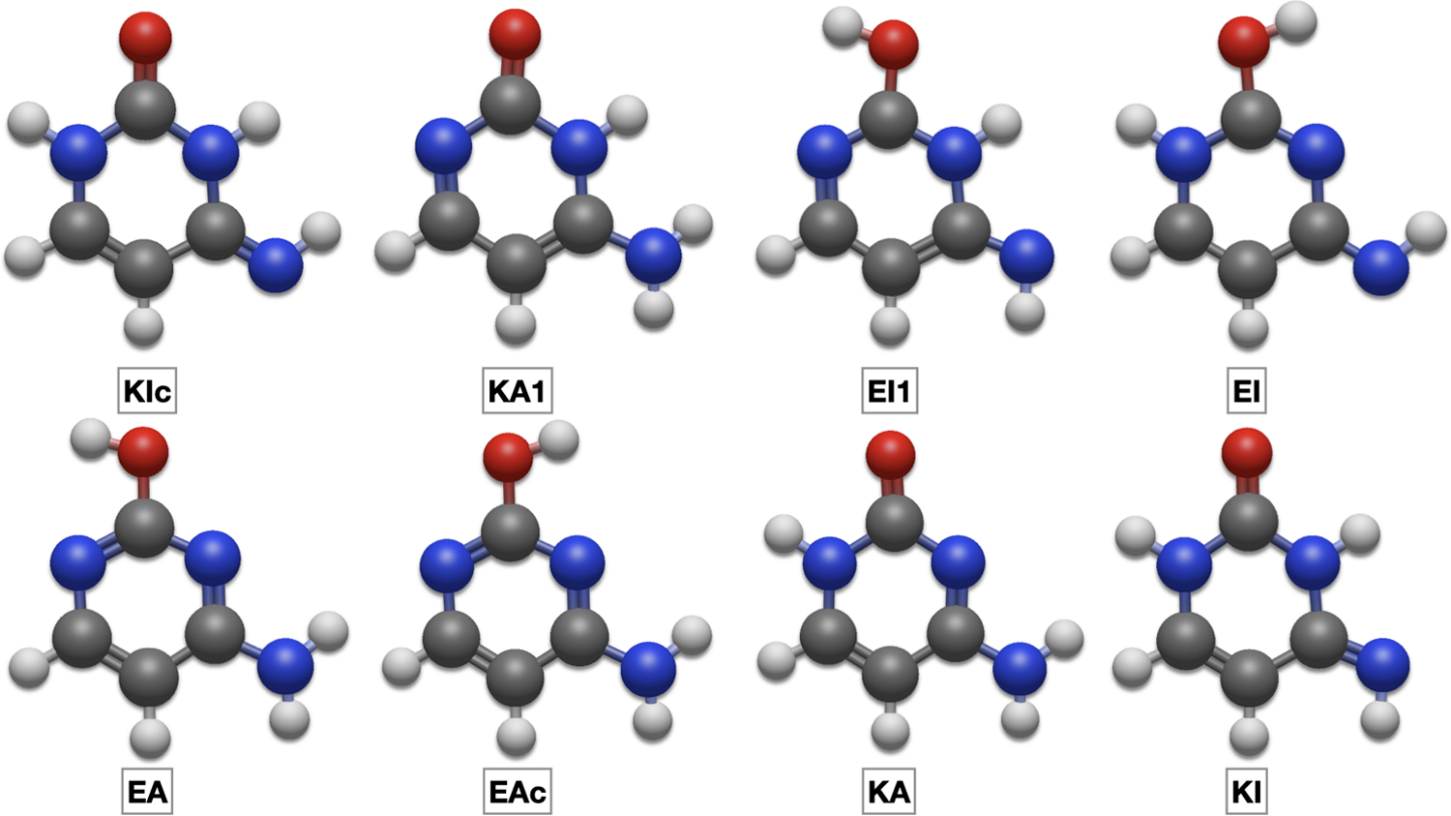
Structure of cytosine tautomers in order of relative stability.

The last benchmark of this section is the tautomeric equilibrium of cytosine (Figure 2), which is quite sensitive to the quality of the employed QC method. As a matter of fact, DFT approaches overstabilize the KA tautomer, whereas the EA tautomer becomes too stable at the MP2 level.^54^ Only CCSD(T) approaches, including both CBS and CV contributions, are able to deliver accurate values. In fact, the most accurate experimental estimate of the relative stability of different tautomers (obtained from the relative intensity of several IR bands)^81^ indicates, in agreement with PCS results, that the EA form is favored in the gas phase and that KA and EAc forms have a (slightly lower) comparable stability. Table 5 shows that junChS and PCS models deliver comparable results, but the contribution of CBS extrapolation is strongly reduced in the latter case (for instance, from 1.5 to 0.7 kJ mol^–1^ for the KA tautomer). Since the accuracy of this contribution is limited due to its evaluation at the MP2 level, its reduction increases the robustness of the additive approach underlying any composite scheme. Finally, the remarkable agreement between PCS and PCS-F12 results (within 0.5 kJ mol^–1^) confirms that CBS extrapolation at the MP2 level is very effective.

**Table 5:** Relative Electronic Energies of All of the Tautomers and Rotamers of Cytosine Computed by Different Methods with PCS/DFT Geometries^a^

tautomer	CC/j3	junChS	PCS	PCS-F12
EA	0.0	0.0	0.0	0.0
EAc	2.9	3.0	3.0	3.0
KA	4.7	3.2	3.1	3.1
KI	7.0	6.1	6.1	6.2
KIc	14.0	13.4	13.2	13.5
KA1	35.0	34.5	34.5	34.4
EI1	51.4	51.5	51.6	52.0
EI	69.6	69.8	69.8	70.3
MAX^b^	1.6	0.5	0.5	
MUE^b^	0.7	0.2	0.2	

a CC stands for CCSD(T), and all of the numerical values are in kJ mol^–1^.

b With respect to PCS-F12 computations.

### 3.4 Geometrical Parameters and Harmonic Frequencies

The PCS/DFT geometries are obtained by adding MP2 CV correlation contributions to the rDSD/3F12 results. The validation of those geometries has been performed by comparison with accurate semiexperimental (SE) equilibrium geometries of a subset (SE32) of the SE127 database^82^ containing the following 32 molecules: CH_4_, CO_2_, HCN, HNC, H_2_O, NH_3_, C_2_H_2_, C_2_H_4_, H_2_CO, CH_2_O_2_ (t-HCCOH), CH_2_NH, BH_3_NH_3_, C_2_H_4_O (Oxirane), C_2_H_4_NH (Aziridine), CH_2_F_2_, HOF, CH_2_CHF, cyc-C_3_H_6_, CH_2_CHF, NH_2_OH, BH_2_OH, HNO, H_2_O_2_,^83^ SO_2_, H_2_S, PH_3_, H_2_CS, CH_2_PH, C_2_H_4_S (Thiirane), H_2_S_2_, HClCO, CH_2_Cl_2_, and CH_2_ClF.

The error statistics for the different models are collected in Table 6, whereas the complete results are given in Tables S5 and S6 in the SI. In general terms, it is confirmed that the rDSD functional delivers geometries significantly more accurate than those obtained by hybrid density functionals and, indeed, comparable to those issued from CCSD(T) computations in conjunction with augmented triple-zeta basis sets. Furthermore, both basis set extension from j3 to 3F12 and CV correlation have a negligible effect on valence and dihedral angles but significantly improve the bond length MUEs.^24,71^ The final accuracy of the PCS/DFT results is not far from that of the (much more expensive) junChS reference, so accurate geometries can also be obtained for quite large molecules and confidently used even for the most demanding applications (e.g., rotational constants, which are the leading terms of rotational spectra^24,49^).

**Table 6:** Maximum Difference (MAX), Mean Unsigned Error (MUE), and Root-Mean-Square Deviation (RMSD) of Geometrical Parameters Computed by B3LYP^84^/j3, M062X^85^/j3, rDSD/j3, PCS/DFT, CCSD(T)/j3 (Indicated as CC), and junChS Levels with Respect to SE Values of the SE32 Set^a^

	B3LYP/j3	M062X/j3	rDSD/j3	PCS/DFT	CC/j3	CC/j3+CV2	junChS
second-row (22 molecules)							
MAX(*r*)	0.0136	0.0356	0.0093	0.0058	0.0112	0.0067	0.0042
MAX(θ, φ)	1.25	1.83	0.69	0.71	0.64	0.65	1.52
MUE(*r*)	0.0020	0.0028	0.0018	0.0011	0.0027	0.0017	0.0006
MUE(θ, φ)	0.18	0.21	0.07	0.09	0.07	0.06	0.07
RMSD(*r*)	0.0045	0.0095	0.0035	0.0024	0.0053	0.0034	0.0014
RMSD(θ, φ)	0.54	0.68	0.23	0.26	0.24	0.19	0.29
third-row (10 molecules)							
MAX(*r*)	0.0227	0.0119	0.0113	0.0057	0.0209	0.0156	0.0020
MAX(θ, φ)	0.82	0.44	0.38	0.24	0.29	0.34	0.10
MUE(*r*)	0.0073	0.0031	0.0044	0.0018	0.0065	0.0038	0.0006
MUE(θ, φ)	0.35	0.15	0.13	0.12	0.10	0.11	0.04
RMSD(*r*)	0.0102	0.0048	0.0051	0.0024	0.0082	0.0054	0.0007
RMSD(θ, φ)	0.42	0.20	0.16	0.14	0.12	0.13	0.05

a Bond lengths in Å, valence, and dihedral angles in degrees.

In the case of harmonic frequencies, omission of CV correlation leads to better results due to an effective error compensation with the neglect of higher-order terms^86^ so that rDSD/3F12 values can be directly employed. The validation of those values has been performed with reference to the following 28 molecules (H28 set) for which accurate reference harmonic frequencies are available: H_2_O,^87^ HCN,^88^ CO_2_,^89^ C_2_H_2_,^90−92^ CO,^93^ HF,^93^ N_2_,^93^ N_2_O,^94^ H_2_,^95^ OH^•^,^95^ Cl_2_,^93^ ClCN,^96^ ClF,^97,98^ CS,^93^ HCl,^93^ SiO,^98^ NH_3_,^99^ CF_2_,^100^ H_2_CO,^101^ C_2_H_2_O,^102^ CH_4_,^103^ HNO,^104^ PH_3_,^105^ CCl_2_,^106^ H_2_CS,^107^ C_2_H_4_,^108^ BH_3_,^109^ and HOCl.^110^ Also in this case, the error statistics are reported in the main text (Table 7), whereas the complete results are given in Tables S7 and S8 in the SI.

**Table 7:** Maximum Difference (MAX), Mean Unsigned Error (MUE), and Root-Mean-Square Deviation (RMSD) of Harmonic Vibrational Frequencies (in cm^–1^) by CCSD(T)-F12b/j3 (Indicated as CC-F12), rDSD/j3, and rDSD/3F12 Computations

	CC-F12/j3	rDSD/j3	rDSD/3F12
second-row (18 molecules)			
MAX	11.2	46.5	31.6
MUE	3.5	6.3	6.6
RMSD	4.3	9.4	9.3
third-row (10 molecules)			
MAX	12.3	22.2	17.4
MUE	3.5	8.4	8.4
RMSD	5.0	10.5	9.6

The RMSD of the reference CCSD(T)-F12b/j3 model is about 5 cm^–1^ for the whole H28 set, with this value being largely sufficient for the quantitative analysis of IR and Raman spectra. Also in this case, the rDSD functional performs a respectable job, with its RMSD (about 10 cm^–1^) being sufficient for most spectroscopic investigations and, above all, for the computation of zero-point energies and partition functions.^13,75^

### 3.5 Zero-Point Energies and Thermodynamic Functions

Zero-point and finite temperature contributions are usually obtained by low-level DFT computations in the framework of the RRHO model, possibly employing empirical scale factors.^111−114^ However, the availability of a resonance-free expression for anharmonic ZPEs,^115,116^ black-box procedures for the smoothing of fundamental frequencies,^15^ and the detection/treatment of hindered rotations (HR)^59^ give access to an automated evaluation of anharmonic ZPEs and vibrational frequencies. Then, vibrational partition functions can be effectively computed employing anharmonic ZPEs and fundamental frequencies (Δ_*i*_) in the standard analytical harmonic expression
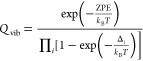
9Equation 9 (referred to as simple perturbation theory, SPT) leads to the same analytical expressions of thermodynamic functions issued from the harmonic-oscillator model and provides results in remarkable agreement with accurate reference values.^58^

In the present context, the reliability of PCS/DFT geometries and semidiagonal fourth-order force fields is analyzed with reference to the accurate ZPEs and entropies available for 21 closed-shell and 10 open-shell semirigid molecules.^13^ The results collected in Tables 8 and 9 show that very accurate results can be obtained for semirigid molecules.

**Table 8:** ZPEs (in kJ mol^–1^) and Entropies at 298 K and 1 atm (in J (mol K)^−1^) for 21 Representative Closed-Shell Molecules

molecule	ZPE_exp_	ZPE_harm_	ZPE_VPT2_	*S*_exp_	*S*_harm_	*S*_SPT_
HF	24.5	24.6	24.4	173.8	173.6	173.5
HCl	17.9	17.9	17.8	186.9	186.6	186.6
H_2_	26.1	26.5(26.1)	26.2	130.6	130.3	130.3
N_2_	13.9	14.0	13.9	191.6	191.6	191.6
F_2_	5.9	5.9	5.9	202.8	202.4	202.4
CO	12.9	13.0	12.9	197.7	197.7	197.7
Cl_2_	3.4	3.4	3.4	223.1	222.7	222.6
CO_2_	30.3	30.5	30.4	213.8	213.8	213.8
CS_2_	18.2	18.2	18.2	238.0	237.8	237.8
H_2_O	55.4	56.2(55.4)	55.3	188.8	188.7	188.6
H_2_S	39.6	40.1(39.3)	39.6	205.8	205.7	205.5
HOF	36.2	36.6(36.2)	36.0	226.8	226.5	226.5
HOCl	34.3	34.8(34.4)	34.3	236.5	236.3	236.2
N_2_O	28.5	28.7	28.5	220.0	219.9	219.9
HCN	41.7	42.1(41.7)	41.7	201.8	201.5	201.6
SO_2_	18.0	18.1	18.0	248.3	248.4	248.3
C_2_H_2_	69.3	70.1(69.3)	69.4	200.9	200.5	200.4
H_2_CO	69.2	70.1(69.3)	69.1	219.0	218.7	218.6
NH_3_	89.0	90.4(89.2)	88.8	192.8	192.4	192.5
CH_4_	116.4	117.8(116.2)	116.1	186.2	186.1	186.1
C_2_H_4_	132.6	134.0(132.4)	132.3	219.3	219.1	219.0
MAX		1.5 (0.3)	0.2		0.5	0.5
MUE		0.4 (0.1)	0.1		0.2	0.3
RMSD		0.6 (0.1)	0.1		0.2	0.3

**Table 9:** ZPEs (in kJ mol^–1^) and Entropies at 298 K and 1 atm (in J (mol K)^−1^) for 10 Representative Open-Shell Molecules

molecule	ZPE_exp_	ZPE_harm_	ZPE_VPT2_	*S*_exp_	*S*_harm_	*S*_SPT_
OH(^2^π)^a^	22.1	22.5(22.1)	22.3	183.7	184.0	183.9
SH(^2^π)^a^	16.0	16.2(15.8)	16.1	195.8	197.9	197.8
CN(^2^σ^+^)^b^	12.4	11.9		202.8	202.7	
NO(^2^π)^a^	11.3	11.6	11.7	210.8	211.1	211.0
NH_2_(^2^B_1_)	48.2	50.5(49.7)	49.7	194.9	194.6	194.5
HCO (^2^A’)	33.9	34.3(33.9)	33.7	224.7	224.3	224.2
HO_2_(^2^A”)	36.8	37.6(37.2)	37.0	229.3	228.8	228.7
CH_3_(^2^A”)	77.4	78.5(77.7)	77.8	194.2	194.8	193.5
t-HOCO(^2^A’)	54.8	55.2(54.8)	54.4		251.5	251.7
CH_3_CO(^2^A’)	111.7	113.8(112.6)	112.2	267.6	268.8	270.2
MAX		2.2 (1.5)	1.5^c^		2.1^d^	2.5^e^
MUE		0.8 (0.4)	0.4^c^		0.6^d^	0.9^e^
RMSD		1.1 (0.6)	0.6^c^		0.9^d^	1.2^e^

a The ZPEs are calculated taking into account the proper degeneracy of the electronic state.

b Restricted open shell with an equilibrium bond length of 1.177 Å, ZPE_VPT2_ are not available for restricted open-shell calculations. The unrestricted results are ZPE_harm_ = 14.4 kJ mol^–1^ and *S*_harm_ = 202.4 J (mol K)^−1^, with ⟨*S*^2^⟩ = 0.853 and an equilibrium bond length of 1.157 Å.

c The CN open-shell molecule is not included in the error analysis.

d The t-HOCO open-shell molecule is not included in the error analysis.

e The CN and t-HOCO open-shell molecules are not included in the error analysis.

However, entropy is strongly sensitive to low-frequency vibrations: as a consequence, small-amplitude vibrations should be treated at the VPT2 level and low-frequency torsions by the hindered-rotor model.

The results collected in Table 10 show that the resulting hindered-rotor/anharmonic-oscillator model (HRAO) performs a remarkable job for systems containing a single torsion, and the same model can be employed in the presence of several loosely coupled large-amplitude motions.

**Table 10:** Absolute Entropies at 298 K and 1 atm (in J (mol K)^−1^) for Representative Molecules Containing One Large-Amplitude Torsion

molecule	*S*_exp_	*S*_harm_	*S*_SPT_
CH_3_CH_3_	229.2	227.5	228.9
CH_3_OH	239.7	238.5	240.0
CH_3_SH	255.1	253.4	255.2
CH_3_CHO	263.8	262.2	264.0
CHOCHO	272.4	271.7	272.3
MAX		1.7	0.3
MUE		1.4	0.2

While more advanced models would be required for strongly coupled large-amplitude motions, sufficiently accurate results can be obtained in most cases by the so-called quasi-harmonic approximation,^117^ which employs a smoothing function to interpolate between free-rotor and harmonic-oscillator partition functions around a critical frequency (about 100 cm^–1^).

### 3.6 Reaction Rate Constants

The accuracy of molecular structures, thermochemical parameters, and energy barriers discussed in the preceding sections suggest that reliable reaction rate constants can be computed whenever the transition state theory (TST)^118^ provides a sound theoretical background. This was indeed demonstrated for a number of reactions in the case of the junChS model,^13,119^ and the PCS results are very similar (generally slightly improved). Therefore, we focus our attention on two additional items, namely, the treatment of quantum effects on the reaction coordinate (e.g., tunneling and nonclassical reflection^120,121^) and the minimization of recrossing trajectories by means of the variational optimization of the transition state (VTST).^122,123^ Furthermore, barrierless elementary steps often come into play in the study of astrochemical reactions or combustion processes. All of those features require additional QC computations, so in the following, we will employ the small curvature tunneling (SCT) model^124^ and the phase space theory (PST),^125^ which usually deliver sufficiently accurate results by means of a limited number of additional computations.

**Figure 3:**
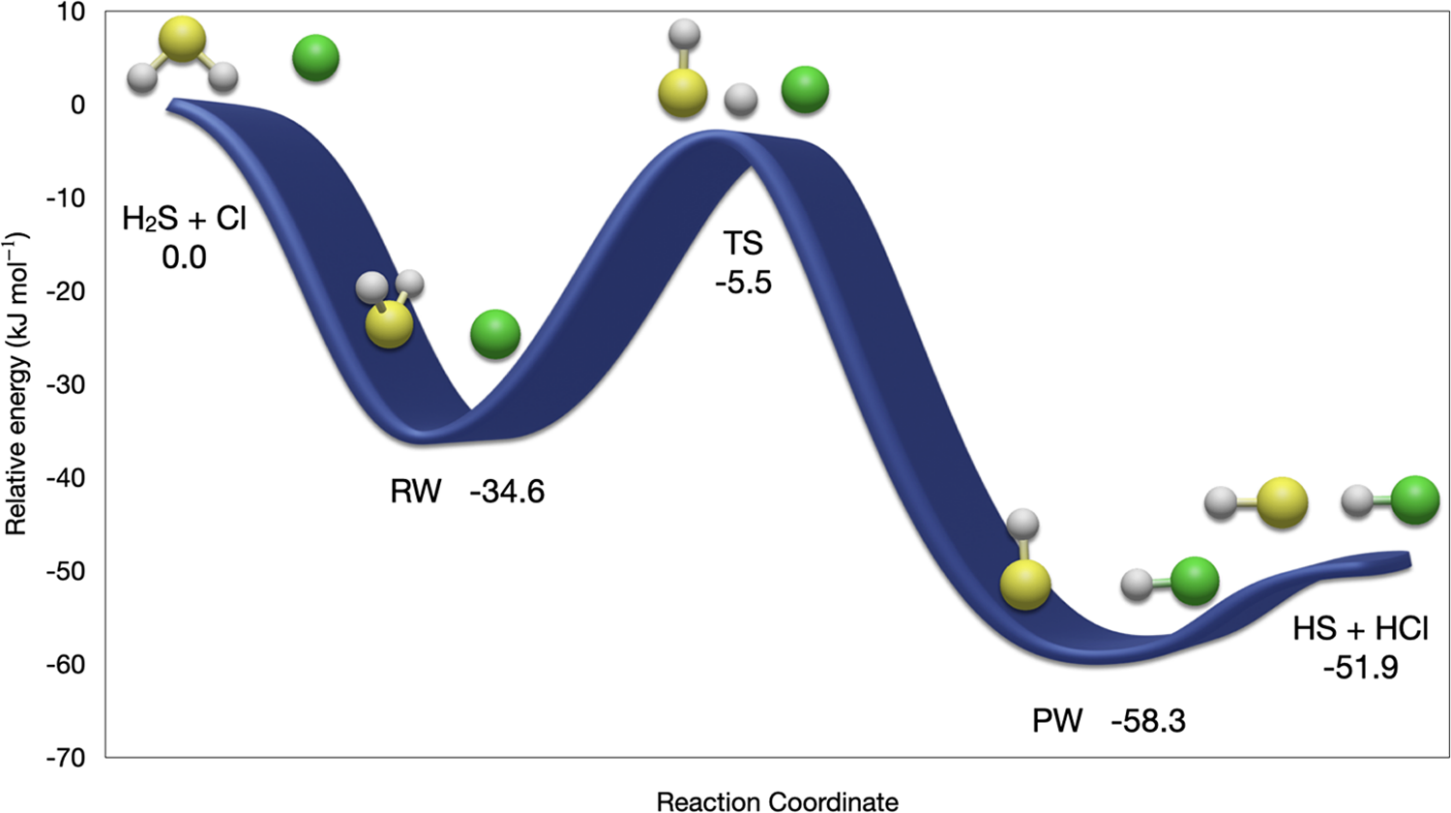
PCS potential energy profile for the H_2_S + Cl → HS + HCl hydrogen abstraction reaction including anharmonic ZPEs (kJ mol^–1^).

The H_2_S + Cl reaction has been chosen as a case study and its overall rate constant has been evaluated employing PCS data in the framework of the master equation approach with the help of the MESS program.^126^ The reaction involves the sequence of elementary steps sketched in Figure 3: the initial interaction between hydrogen sulfide (H_2_S) and chlorine (Cl), driven by noncovalent forces leads to a prereactive complex labeled RW.

Then, under atmospheric conditions, the only open reaction channel follows an addition/elimination mechanism. In detail, after isomerization of the RW complex governed by the transition state (TS), the reaction proceeds toward the final van der Waals complex (PW), which is stabilized by an HS-HCl hydrogen bond. Dissociation of this complex gives the final products of hydrogen abstraction, namely, HS and HCl. The overall rate constant is ruled by the conversion of RW into PW and by the formation of RW, whereas the decomposition of PW plays a negligible role since it is faster than the other two steps.

The relative electronic energies obtained by different computational approaches for the stationary points governing the reaction are collected in Table 11. Extensive benchmarks showed that the role of triple and quadruple excitations is quite limited, whereas CV correlation cannot be neglected for obtaining quantitative results. Noted is that contributions of comparable magnitude are provided by the difference between harmonic and anharmonic ZPEs. Finally, spin–orbit contributions are non-negligible only for reactants (3.3 kJ mol^–1^) and products (2.1 kJ mol^–1^). It is quite apparent that the PCS model chemistry performs a remarkable job (errors generally within 0.5 kJ mol^–1^ with respect to the most accurate results) in view of its limited computational cost and lack of empirical parameters. The only non-negligible difference from the most accurate results concerns the relative stability of RW, which is underestimated by about 2 kJ mol^–1^.

**Table 11:** Relative Electronic Energies for the Stationary Points of the Reaction H_2_S + Cl Obtained by Different Methods^a^

	reactants	RW	TS	PW	products
level of theory	H_2_S + Cl	H_2_S···Cl	HS···H···Cl	HS···HCl	HS + HCl
CBS + CV	0.0	–43.9	–0.1	–57.0	–46.5
Heat-like^119^	0.0	–41.9	0.0	–56.8	–46.2
PCS	0.0	–40.1	0.4	–56.9	–46.3
ΔZPE_h_	0.0	5.9	–5.3	–1.6	–5.9
ΔZPE_a_	0.0	5.5	–5.9	–1.4	–5.6

a CBS+CV refers to CCSD(T) computations including complete basis set (CBS) extrapolation and core–valence (CV) correlation computed at the same level, whereas Heat-like refers to CCSDTQ results, together with CBS extrapolation and CV correlation at the CCSD(T) level, also scalar relativistic effects, diagonal Born–Oppenheimer, and spin–orbit contributions. rDSD/j3 harmonic (ΔZPE_h_) and anharmonic (ΔZPE_a_) ZPE contributions are also given. All of the values are in kJ mol^–1^.

It could be argued that the geometries employed in all of the computations (optimized at the PCS/DFT level) could reduce the overall accuracy of the results, but reoptimization of the geometries at the PCS level leads to average differences within 0.002 Å for bond lengths and 0.2° for angles. Once again, the largest discrepancy is observed for RW (overestimation of 0.01 Å for the S–Cl distance), but the relative energies computed at the PCS level employing PCS/DFT or PCS geometries never differ by more than 0.2 kJ mol^–1^. In summary, PCS energies are sufficiently accurate to be confidently used to characterize the energetics of the reaction, employing geometrical structures optimized at the PCS/DFT level.

The overall rate constant has been computed employing the PST for the entrance and exit channels together with the VTST model in conjunction with the SCT approximation^121^ for the step involving a distinct TS. Noted is that previous computations showed that the error of PST with respect to more accurate multidimensional computations is within 20% and that one degree of freedom needs to be treated as a hindered rotor at the TS.^119^ The computed rate constant changes between 1.22 × 10^–10^ and 4.30 × 10^–11^ cm^3^ mol^–1^ s^–1^ when going from 200 to 900 K, whereas the corresponding experimental value changes between 1.06 × 10^–10^ and 3.95 × 10^–11^ cm^3^ mol^–1^ s^–1^ . We can conclude that the master equation approach based on PCS data also performs a remarkable job for this quite challenging reaction, which is representative of complex mechanisms involving both distinct transition states and barrierless steps.

## Timings, Accuracy, and Extension to Larger Molecules

The choice of the computational levels for the different terms included in the PCS model has been based on the requirement of comparable resources for each of them with specific reference to molecules containing up to about 20 atoms. In particular, the cost of CCSD(T)/2F12 and MP2/4F12 computations is negligible with respect to their CCSD(T)/3F12 counterparts, whose cost is, in turn, comparable with that of rDSD/3F12+CV2 geometry optimizations, rDSD/3F12 harmonic frequencies, and B3/SVP anharmonic computations. The effective implementation of all these contributions in the Gaussian computer code allowed us to perform all of the computations reported in the previous sections within reasonable times employing a standard workstation. Furthermore, thanks to the use of the 3F12 basis set and two-point extrapolation, conventional computations provide results close to those of their explicitly correlated counterparts without the need for any empirical parameter. In this connection, together with the results presented in the previous sections, a recent study of guanine tautomers^31^ showed that PCS results are very close to the best available values,^127^ which were obtained at the W1–F12 level.^7^

For larger systems, containing up to about 50 atoms, single-point CCSD(T) and CCSD(T)-F12 energy evaluations with the j3 or 3F12 basis sets can be routinely performed thanks to efficient parallelization and implementation of frozen natural orbital (FNO), natural auxiliary functions (NAF) and/or related reduced-scaling techniques in several computer codes.^128−133^ For purposes of illustration, the relative stabilities of cytosine tautomers have been recomputed with both the FNO–CCSD(T)^133^ and FNO–CCSD(F12*)(T+)^134^ models employing the standard thresholds of the MRCC code. The results collected in Table 12 show that FNO (and related) approximations produce MUEs of 0.3 and 0.1 kJ mol^–1^ from full computations, which are accompanied by reductions of computer times of about 10 and 5 times for conventional and explicitly correlated models, respectively.

**Table 12:** Relative Electronic Energies of All of the Tautomers and Rotamers of Cytosine Computed by Different Methods with PCS/DFT Geometries^a^

tautomer	PCS	FNO-PCS	PCS-F12	FNO-PCS-F12
EA	0.0	0.0	0.0	0.0
EAc	3.0	2.8	3.0	2.9
KA	3.1	3.3	3.1	3.0
KI	6.1	5.8	6.2	6.3
KIc	13.2	12.9	13.5	13.5
KA1	34.5	34.4	34.4	34.5
EI1	51.6	51.2	52.0	52.0
EI	69.8	69.3	70.3	70.3
MAX^b^		0.5		0.1
MUE^b^		0.3		0.1

a All of the numerical values are in kJ mol^–1^.

b FNO-PCS and FNO-PCS-F12 with respect to PCS and PCS-F12, respectively.

Above 40–50 atoms, local approximations start to be competitive and lead to an essentially linear increase of computer time with the dimensions of the investigated system.^135−137^ However, the accuracy of these methods for the most demanding applications needs further investigation.^138,139^

The situation is different for gradient evaluations, where fast implementations are available only for DFT and MP2 (hence double-hybrids). Therefore, PCS/DFT represents an attractive approach, and a further reduction of computational resources is obtained by neglecting d functions on first-row atoms and replacing the two f functions on second- and third-row atoms with a single f function taken from the cc-pVTZ basis set.^70^ Furthermore, CV corrections are entirely negligible for valence or dihedral angles,^24^ and their contribution to bond lengths (*r*_*ij*_) mainly depends on the principal quantum numbers (*n*_*i*_, *n*_*j*_) of the involved atoms *i* and *j*(24,140)
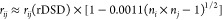
10Since all of these simplifications have a negligible effect on the final accuracy of the results, the introduction of a single empirical parameter leads to the low-cost PCS/Bonds model,^140^ which requires a single DFT geometry optimization with a triple-zeta basis set. In the case of kinetic parameters, this recipe can be employed to trace the intrinsic reaction coordinate along which more accurate energy computations can be performed to locate an improved transition state and the corresponding energy barrier by the so-called IRCmax approach.^141^ Also in this case, molecules containing up to about 50 atoms can be routinely studied.^24,140^ For larger systems the results delivered by hybrid functionals in conjunction with double-zeta basis sets can be further improved by linear regression (LR),^142^ templating molecule (TM),^82,143^ or, possibly, machine learning approaches.

Coming to zero-point energies and entropies, harmonic frequencies obtained at the rDSD/3F12 level do not require any empirical correction to compensate for method and/or basis set deficiency, but only for genuine anharmonic effects.^112,114,144^ The results collected in Tables 8 and 9 show that anharmonic terms give a non-negligible contribution to ZPEs only in the presence of some XH bonds (X = H, C, N, O, S). As a consequence, an empirical correction of 0.4 kJ mol^–1^ for each bond of this kind provides results very close to their anharmonic counterparts for both closed- and open-shell systems (see results in parentheses in Tables 8 and 9). Concerning entropies, harmonic results are already sufficiently reliable, except when hindered rotations are present. In this case, for large molecules one can resort to the black-box quasi-harmonic (QH) approximation^117^ in which, below a given threshold, entropic terms are obtained from the free-rotor model, and a damping function is used to interpolate between free-rotor and harmonic-oscillator expressions near the cutoff frequency. Finally, when also harmonic computations by double-hybrid functionals become too expensive, one can resort to scaled harmonic frequencies^111,113^ computed by hybrid functionals in conjunction with double-zeta basis sets, preferably employing different scaling factors for different ranges of frequencies.^114^

## Conclusions

The main target of the present study was the computation of accurate formation, reaction, and activation energies, together with geometrical parameters, harmonic frequencies, thermodynamic functions, and rate constants, in the framework of a general strategy that aims at the accurate structural and spectroscopic characterization of medium-sized molecules. The main outcome is that accurate electronic energies can be computed by the new PCS model at PCS/DFT geometries. Inclusion of MP2 core–valence correlation and CBS extrapolation for both MP2 and post-MP2 contributions leads to remarkably accurate results, while the availability of reduced cost CCSD(T) implementations, possibly involving local correlation treatments,^135−137^ significantly increases the dimensions of investigable systems. In summary, the way seems to be paved toward the systematic study of physical-chemical processes in the gas phase at a reasonable cost by an accurate black-box procedure.

## Supporting Information Available

Additional data for atomization reaction and activation energies, geometrical parameters, and harmonic frequencies (PDF)
