# Supplementary material for: Accurate Thermochemical and Kinetic Parameters at Affordable Cost by Means of the Pisa Composite Scheme (PCS)
Source: J Chem Theory Comput. 2023 Sep 29;19(20):7273–86. doi: 10.1021/acs.jctc.3c00817 (PMC10601482; doi:10.1021/acs.jctc.3c00817)
Supplement: Supplementary file 1 — ct3c00817_si_001.pdf [file ct3c00817_si_001.pdf]

Supporting Information: Accurate Thermochemical and Kinetic Parameters at Affordable Cost by Means of the Pisa Composite Scheme (PCS)

Vincenzo Barone,[*,†] Luigi Crisci,[†] and Silvia Di Grande[†,‡]

†*Scuola Normale Superiore di Pisa, Piazza dei Cavalieri 7, 56125 Pisa, Italy*

‡*Scuola Superiore Meridionale, Largo San Marcellino 10, 80138 Napoli, Italy*

E-mail: vincenzo.barone@sns.it

1 KAW test set

Table S1: KAW test set[S1] atomization energies ($kJ\,mol^{-1}$) computed at CCSD(T)/aug-cc-pV(5,6)Z (reference energies, Ref. S2), CCSD(T)/3F12, PCS, and junChS calculated with the Gaussian software,[S3] CCSD(F12*)(T+)/3F12, PCS-F12 and junChS-F12 employing CCSD(F12*)(T+) with the MRCC computational package,[S4,S5] while the CCSD(T)-F12b junChS-F12 version with the MOLPRO one[S6,S7]

	Ref.	CCSD(T)/3F12	PCS	junChS	CCSD(F12*)(T+)/3F12	PCS-F12	junChS-F12[a]
F_2	183.2	149.7	161.6	162.5	159.2	160.9	160.3 (159.4)
Cl_2	268.8	228.3	246.7	248.5	247.0	247.1	248.9 (246.8)
ClF	287.4	247.5	261.6	263.0	260.5	260.9	261.3 (259.5)
$ClO^{\bullet}$	270.9	251.9	269.8	268.6	267.1	268.4	267.9 (267.2)
Si_2	327.1	295.1	313.8	311.1	308.9	311.4	310.2 (309.2)
NH	339.0	336.1	348.5	349.1	345.5	346.6	346.0 (345.1)
$CH^{\bullet}$	340.0	343.8	353.1	353.3	350.3	351.2	350.6 (349.9)
S_2	481.8	406.1	430.5	429.9	429.5	430.6	431.3 (428.8)
$OH^{\bullet}$	451.3	436.1	449.0	450.3	446.5	447.8	447.8 (446.4)
HCl	452.3	439.8	449.3	450.2	448.4	448.5	448.8 (447.8)
P_2	509.8	451.6	486.5	484.9	480.9	483.9	483.1 (479.0)
O_2	601.7	477.3	494.8	495.2	492.5	494.4	494.2 (492.5)
SO	593.6	503.7	524.6	522.4	522.5	524.0	523.0 (520.9)
$SiH_2(^3B_1)$	547.1	548.8	559.8	558.8	556.8	557.5	556.1 (555.6)
HF	611.2	579.6	592.8	595.1	591.0	592.0	592.6 (590.8)
$NO^{\bullet}$	689.8	611.2	635.6	636.9	633.0	635.4	634.7 (631.6)
$SiH_2(^1A_1)$	622.4	633.5	647.0	647.7	642.2	643.3	641.8 (641.3)
$PH_2^{\bullet}$	625.8	629.8	647.9	648.3	642.9	644.3	643.0 (641.6)
HOCl	724.7	669.5	695.0	696.6	691.9	693.3	694.0 (690.7)
CS	750.6	695.3	714.3	715.3	714.7	714.3	715.5 (711.6)
$CN^{\bullet}$	792.6	726.6	750.4	750.8	746.5	748.5	747.2 (744.9)
$CH_2(^1A_1)$	739.1	742.2	759.5	760.2	754.7	756.2	755.3 (754.1)
$NH_2^{\bullet}$	755.7	741.8	765.1	766.8	760.1	762.0	761.3 (759.4)
H_2S	758.9	752.4	769.3	770.4	766.4	766.9	766.6 (764.9)
$CH_2(^3B_1)$	795.0	782.7	796.7	796.6	793.7	794.7	794.3 (793.1)
SiO	862.4	779.3	802.6	800.6	800.0	801.7	799.8 (796.9)
N_2	1009.3	920.6	950.2	951.9	947.2	949.6	948.5 (944.7)
$SiH_3^{\bullet}$	935.3	940.9	958.5	959.0	953.3	954.3	952.6 (951.8)
H_2O	992.7	951.5	975.1	978.7	971.3	973.4	973.9 (971.1)
PH_3	984.3	990.6	1015.5	1016.3	1008.9	1010.6	1008.9 (1007.0)
CO	1140.4	1061.1	1081.7	1083.7	1081.1	1082.6	1082.7 (1078.7)
SO_2	1193.5	1035.4	1082.5	1079.2	1079.2	1080.9	1081.2 (1075.2)
H_2O_2	1161.0	1089.4	1126.6	1129.2	1120.2	1123.8	1123.9 (1119.3)
$HCO^{\bullet}$	1215.5	1138.3	1164.6	1165.8	1161.7	1163.9	1163.4 (1159.7)
NH_3	1246.2	1215.7	1247.9	1251.1	1241.6	1244.1	1243.6 (1240.6)
$CH_3^{\bullet}$	1280.5	1264.7	1287.3	1288.5	1282.0	1283.6	1283.1 (1281.3)
HCN	1357.6	1273.1	1305.7	1306.5	1300.6	1303.1	1302.0 (1297.7)
SiH_4	1332.8	1340.9	1364.5	1366.5	1357.0	1358.3	1356.6 (1355.6)
H_2CO	1605.0	1531.1	1564.3	1566.1	1559.6	1562.2	1561.9 (1557.6)
CO_2	1735.6	1585.8	1623.3	1626.1	1622.5	1625.3	1625.9 (1618.5)
CH_3Cl	1669.3	1622.2	1655.8	1656.6	1650.5	1651.8	1652.0 (1647.7)
C_2H_2	1732.5	1655.5	1690.2	1690.8	1683.8	1686.7	1685.4 (1681.1)
CH_4	1748.1	1729.4	1758.9	1760.2	1751.9	1753.7	1753.2 (1750.8)
N_2H_4	1845.0	1778.0	1834.9	1839.3	1824.6	1828.9	1827.9 (1821.9)
CH_3SH	1989.8	1947.0	1987.5	1988.9	1980.6	1982.1	1981.7 (1976.8)
CH_3OH	2170.8	2102.5	2147.9	2150.3	2139.8	2143.2	2143.0 (2137.7)
Si_2H_6	2206.3	2206.1	2251.5	2252.0	2238.0	2241.1	2237.2 (2234.5)
C_2H_4	2369.7	2313.7	2355.7	2357.6	2347.9	2350.5	2349.7 (2345.2)
C_2H_6	2981.6	2929.8	2981.5	2983.4	2970.7	2973.8	2972.9 (2967.7)

[a] CCSD(F12*)(T+) and, in parenthesis, CCSD(T)-F12b from Ref. S8.

Table S2: KAW test set[S1] closed-shell reaction energies ($kJ\,mol^{-1}$) computed at CCSD(T)/aug-cc-pV(5,6)Z (reference energies, Ref. S2), CCSD(T)/3F12, PCS, and junChS calculated with the Gaussian software,[S3] CCSD(F12*)(T+)/3F12, PCS-F12 and junChS-F12 employing CCSD(F12*)(T+) with the MRCC computational package,[S4,S5] while the CCSD(T)-F12b junChS-F12 version with the MOLPRO one[S6,S7]

.

	Ref.	CCSD(T)/3F12	PCS	junChS	CCSD(F12*)(T+)/3F12	PCS-F12	junChS-F12[a]
$CO + H_2 \longrightarrow H_2CO$	-23.8	-16.6	-22.7	-21.4	-21.3	-22.1	-21.4 (-21.0)
$CO + H_2O \longrightarrow CO_2 + H_2$	-43.4	-26.7	-26.4	-24.6	-27.4	-26.8	-27.2 (-26.5)
$CH_3OH + HCl \longrightarrow CH_3Cl + H_2O$	-38.9	-31.4	-33.8	-34.8	-33.6	-33.6	-34.1 (-33.3)
$H_2O + CO \longrightarrow HCOOH$	-42.7	-32.7	-38.6	-35.5	-37.6	-38.0	-37.5 (-36.6)
$CH_3OH + H_2S \longrightarrow CH_3SH + H_2O$	-52.9	-43.6	-45.4	-46.9	-45.7	-45.5	-46.0 (-45.3)
$CS_2 + 2\,H_2O \longrightarrow CO_2 + 2\,H_2S$	-19.9	-58.6	-47.5	-45.1	-48.1	-48.1	-45.2 (-47.2)
$C_2H_6 + H_2 \longrightarrow 2\,CH_4$	-73.7	-75.5	-76.3	-76.0	-75.8	-76.1	-75.7 (-76.1)
$HNCO + H_2O \longrightarrow CO_2 + NH_3$	-85.7	-87.6	-85.5	-84.9	-86.2	-86.0	-85.7 (-86.1)
$CH_4 + Cl_2 \longrightarrow CH_3Cl + HCl$	-104.7	-104.4	-99.5	-98.0	-100.0	-99.5	-98.8 (-97.9)
$Cl_2 + F_2 \longrightarrow 2\,ClF$	-122.8	-117.0	-115.0	-114.9	-114.8	-113.7	-113.5 (-112.8)
$CO + Cl_2 \longrightarrow COCl_2$	-136.9	-114.2	-116.3	-114.3	-116.8	-115.6	-116.4 (-113.7)
$CO_2 + 3\,H_2 \longrightarrow CH_3OH + H_2O$	-105.4	-107.9	-119.9	-120.0	-116.9	-118.8	-117.5 (-116.9)
$HCHO + H_2 \longrightarrow CH_3OH$	-124.9	-118.0	-123.6	-123.2	-123.0	-123.5	-123.3 (-122.3)
$CO + 2\,H_2 \longrightarrow CH_3OH$	-148.7	-134.6	-146.3	-144.6	-144.3	-145.5	-144.7 (-143.4)
$C_2H_4 + H_2 \longrightarrow C_2H_6$	-171.1	-162.7	-165.8	-164.8	-165.6	-165.8	-165.3 (-164.7)
$SO_3 + CO \longrightarrow SO_2 + CO_2$	-189.0	-188.0	-180.7	-183.9	-181.9	-181.8	-182.5 (-181.7)
$H_2 + Cl_2 \longrightarrow 2\,HCl$	-195.0	-197.9	-191.9	-190.9	-192.5	-192.4	-191.0 (-190.9)
$C_2H_2 + H_2 \longrightarrow C_2H_4$	-196.4	-204.8	-205.6	-205.8	-206.8	-206.3	-206.5 (-206.2)
$SO_2 + H_2O_2 \longrightarrow SO_3 + H_2O$	-237.9	-198.8	-209.5	-208.0	-210.7	-210.5	-210.9 (-209.8)
$CO + 3\,H_2 \longrightarrow CH_4 + H_2O$	-277.9	-259.5	-272.5	-272.2	-270.4	-272.0	-271.0 (-269.7)
$HCN + 3\,H_2 \longrightarrow CH_4 + NH_3$	-314.1	-311.8	-321.3	-321.8	-321.1	-322.2	-321.4 (-320.2)
$H_2O_2 + H_2 \longrightarrow 2\,H_2O$	-383.5	-360.1	-363.7	-367.3	-365.2	-365.5	-366.1 (-365.0)
$CO + H_2O_2 \longrightarrow CO_2 + H_2O$	-426.9	-386.8	-390.2	-391.9	-392.6	-392.3	-393.3 (-391.5)
$2\,NH_3 + 3\,Cl_2 \longrightarrow N_2 + 6\,HCl$	-424.3	-443.1	-409.9	-405.3	-413.4	-411.1	-407.8 (-409.8)
$3\,N_2H_4 \longrightarrow 4\,NH_3 + N_2$	-459.1	-449.4	-437.1	-438.3	-439.6	-439.2	-439.2 (-441.4)
$H_2 + F_2 \longrightarrow 2\,HF$	-598.4	-556.0	-564.0	-566.7	-565.6	-565.5	-567.0 (-564.4)
$CH_4 + 4\,H_2O_2 \longrightarrow CO_2 + 6\,H_2O$	-1299.6	-1207.6	-1208.9	-1221.5	-1217.9	-1216.8	-1220.7 (-1216.7)
$2\,NH_3 + 3\,F_2 \longrightarrow N_2 + 6\,HF$	-1634.5	-1517.3	-1526.3	-1532.9	-1532.7	-1530.5	-1535.9 (-1530.2)

[a] CCSD(F12*)(T+) and, in parenthesis, CCSD(T)-F12b from Ref. S8.

Table S3: KAW test set[S1] open-shell reaction energies ($kJ\,mol^{-1}$) computed at CCSD(T)/aug-cc-pV(5,6)Z (reference energies, Ref. S2), CCSD(T)/3F12, PCS, and junChS calculated with the Gaussian software,[S3] CCSD(F12*)(T+)/3F12, PCS-F12 and junChS-F12 employing CCSD(F12*)(T+) with the MRCC computational package,[S4,S5] while the CCSD(T)-F12b junChS-F12 version with the MOLPRO one[S6,S7]

	Ref.	CCSD(T)/3F12	PCS	junChS	CCSD(F12*)(T+)/3F12	PCS-F12	junChS-F12[a]
$HCl + H^\bullet \longrightarrow Cl^\bullet + H_2$	11.5	-13.6	-10.7	-10.8	-8.8	-9.0	-9.0 (-10.0)
$H_2O + F_2 \longrightarrow 2\,HF + O$	-46.5	-57.9	-48.8	-49.0	-51.6	-49.6	-51.0 (-51.1)
$CH_4 + OH^\bullet \longrightarrow CH_3^\bullet + H_2O$	-73.9	-50.8	-54.6	-56.7	-54.9	-55.6	-55.9 (-55.1)
$OH^\bullet + H_2 \longrightarrow H_2O + H^\bullet$	-100.6	-62.0	-66.2	-67.4	-67.5	-68.1	-68.3 (-66.8)
$CO + OH^\bullet \longrightarrow CO_2 + H^\bullet$	-143.9	-88.7	-92.6	-92.0	-94.9	-94.9	-95.5 (-93.3)
$CH_3^\bullet + Cl_2 \longrightarrow CH_3Cl + Cl^\bullet$	-120.0	-129.2	-121.8	-119.6	-121.5	-121.1	-120.1 (-119.6)
$S + 2\,HCl \longrightarrow H_2S + Cl_2$	-123.1	-101.0	-117.5	-118.6	-116.6	-117.0	-117.8 (-116.2)
$2\,NO^\bullet + O_2 \longrightarrow 2\,NO_2^\bullet$	-137.6	-111.1	-124.1	-125.6	-125.5	-125.4	-127.1 (-122.8)
$N + O_2 \longrightarrow NO^\bullet + O$	-88.1	-133.9	-140.9	-141.7	-140.5	-141.0	-140.5 (-139.2)
$4\,HCl + O_2 \longrightarrow 2\,H_2O + 2\,Cl_2$	-112.1	-122.9	-151.9	-158.5	-150.6	-152.7	-156.0 (-152.2)
$NO^\bullet + H_2O_2 \longrightarrow NO_2^\bullet + H_2O$	-201.3	-156.3	-158.0	-159.9	-160.2	-159.5	-160.7 (-159.3)
$2\,NO^\bullet \longrightarrow N_2 + O_2$	-231.4	-175.4	-173.7	-173.3	-173.8	-173.2	-173.3 (-173.9)
$2\,H_2O_2 \longrightarrow 2\,H_2O + O_2$	-265.0	-201.4	-191.8	-194.2	-194.8	-193.5	-194.3 (-195.9)
$Cl_2 + H^\bullet \longrightarrow HCl + Cl^\bullet$	-183.5	-211.5	-202.6	-201.7	-201.4	-201.4	-200.0 (-201.0)
$2\,SO_2 + O_2 \longrightarrow 2\,SO_3$	-210.8	-196.2	-227.1	-221.7	-226.6	-227.5	-227.4 (-223.7)
$Cl^\bullet + OH^\bullet \longrightarrow HOCl$	-273.4	-233.5	-246.0	-246.3	-245.4	-245.6	-246.2 (-244.2)
$H_2S + F_2 \longrightarrow S + 2\,HF$	-280.3	-257.1	-254.7	-257.3	-256.5	-256.2	-258.3 (-257.3)
$2\,NH_2^\bullet \longrightarrow N_2H_4$	-333.6	-294.4	-304.7	-305.7	-304.5	-305.0	-305.3 (-303.1)
$NO + N \longrightarrow O + N_2$	-319.5	-309.3	-314.6	-315.0	-314.3	-314.2	-313.8 (-313.1)
$O + 2\,HCl \longrightarrow H_2O + Cl_2$	-356.9	-300.1	-323.3	-326.9	-321.5	-323.5	-325.1 (-322.3)
$2\,SiH_3^\bullet \longrightarrow Si_2H_6$	-335.6	-324.3	-334.5	-334.0	-331.5	-332.4	-332.0 (-330.8)
$CH_3^\bullet + HS^\bullet \longrightarrow CH_3SH$	-348.1	-324.8	-333.0	-333.0	-333.4	-332.8	-333.3 (-331.1)
$SO_2 + O \longrightarrow SO_3$	-406.3	-336.8	-360.9	-358.5	-359.5	-360.9	-360.8 (-358.1)
$CS + O \longrightarrow CO + S$	-389.8	-365.8	-367.4	-368.5	-366.4	-368.3	-367.2 (-367.2)
$CH_3^\bullet + Cl^\bullet \longrightarrow CH_3Cl$	-388.8	-357.5	-368.5	-368.1	-368.5	-368.2	-369.0 (-366.4)
$CH_3OHO + O \longrightarrow HCHO + H_2O$	-426.9	-380.1	-391.6	-394.5	-391.1	-392.4	-392.8 (-390.9)
$CH_3^\bullet + OH^\bullet \longrightarrow CH_3OH$	-439.0	-401.7	-411.5	-411.5	-411.2	-411.8	-412.1 (-410.0)
$NH + H^\bullet \longrightarrow NH_2^\bullet$	-416.7	-405.8	-416.6	-417.7	-414.5	-415.3	-415.2 (-414.2)
$Si + H_2 \longrightarrow SiH_4$	-451.1	-434.1	-444.6	-444.5	-442.5	-443.3	-440.9 (-440.0)
$CS + S \longrightarrow CS_2$	-497.5	-433.7	-449.9	-449.2	-449.9	-449.9	-450.6 (-447.4)
$NH_2^\bullet + H^\bullet \longrightarrow NH_3$	-490.5	-473.9	-482.8	-484.3	-481.5	-482.1	-482.3 (-481.2)
$2\,H_2 + O_2 \longrightarrow 2\,H_2O$	-502.0	-518.8	-535.7	-540.3	-535.7	-537.5	-538.0 (-534.1)
$CO_2 + C \longrightarrow 2\,CO$	-545.2	-536.3	-540.0	-541.4	-539.6	-539.9	-539.4 (-539.0)
$CO + O \longrightarrow CO_2$	-595.2	-524.8	-541.7	-542.4	-541.5	-542.7	-543.3 (-539.7)
$C + H_2O \longrightarrow CO + H_2$	-588.5	-563.0	-566.5	-566.0	-567.0	-566.7	-566.6 (-565.5)
$N_2H_4 + O_2 \longrightarrow N_2 + 2\,H_2O$	-548.0	-568.1	-570.8	-574.9	-572.7	-573.1	-574.3 (-572.5)
$2\,NH \longrightarrow N_2 + H_2$	-772.1	-701.8	-713.1	-714.7	-713.4	-713.8	-714.2 (-712.3)
$C + S_2 \longrightarrow CS_2$	-766.3	-722.8	-733.7	-734.5	-735.1	-733.5	-734.8 (-730.1)
$2\,CO + 2\,NO^\bullet \longrightarrow N_2 + 2\,CO_2$	-820.1	-747.7	-762.3	-762.9	-764.2	-764.2	-765.6 (-760.9)
$CH_4 + 2\,O_2 \longrightarrow CO_2 + 2\,H_2O$	-769.5	-804.7	-825.2	-833.0	-828.3	-829.7	-832.2 (-824.9)
$4\,NH_3 + 5\,O_2 \longrightarrow 4\,NO^\bullet + 6\,H_2O$	-722.2	-904.1	-927.9	-939.8	-931.1	-933.9	-937.0 (-928.4)
$2\,NH_3 + 2\,NO^\bullet + O \longrightarrow 2\,N_2 + 3\,H_2O$	-1124.7	-1041.6	-1058.7	-1064.0	-1059.3	-1060.5	-1062.1 (-1058.2)
$C + O_2 \longrightarrow CO_2$	-1133.9	-1108.5	-1128.6	-1130.9	-1130.0	-1131.0	-1131.8 (-1126.0)
$CS_2 + 3\,O_2 \longrightarrow CO_2 + 2\,SO_2$	-1069.4	-1095.7	-1139.9	-1134.6	-1139.0	-1139.9	-1139.6 (-1132.5)
$CH_4 + 4\,NO^\bullet \longrightarrow 2\,N_2 + CO_2 + 2\,H_2O$	-1232.3	-1155.6	-1172.6	-1179.6	-1175.8	-1176.0	-1178.7 (-1172.7)
$CH_4 + NH_3 + 3\,O \longrightarrow HCN + 3\,H_2O$	-1341.5	-1182.4	-1224.3	-1231.4	-1221.1	-1225.6	-1226.9 (-1219.5)
$2\,C + H_2 \longrightarrow C_2H_2$	-1291.6	-1202.1	-1230.3	-1229.8	-1226.6	-1229.1	-1227.6 (-1223.3)
$4\,NH_3 + 3\,O_2 \longrightarrow 2\,N_2 + 6\,H_2O$	-1185.0	-1255.0	-1275.3	-1286.3	-1278.6	-1280.2	-1283.5 (-1276.2)

[a] CCSD(F12*)(T+) and, in parenthesis, CCSD(T)-F12b from Ref. S8.

Table S4: Maximum difference (MAX), mean unsigned error (MUE), and root-mean-square deviation (RMSD) for the KAW test for CCSD(T)/3F12, CCSD(T)/(2,3)F12 and PCS computations and their explicitly correlated counterparts. All the numerical values are in $\mathrm{kJ\,mol^{-1}}$.

	CCSD(T)/3F12	CCSD(T)/(2,3)F12	PCS	CCSD(F12*)(T+)/3F12	CCSD(F12*)(T+)/(2,3)F12	PCS-F12
Atomization energies						
MAX	53.7	23.7	8.8	9.9	10.2	8.4
MUE	25.0	9.9	2.9	3.7	2.6	2.3
RMSD	27.2	11.0	3.6	4.5	3.8	3.4
Closed-shell react. energies						
MAX	35.7	20.5	7.6	6.0	3.6	3.6
MUE	7.3	4.3	1.6	1.0	1.0	0.8
RMSD	10.0	6.1	2.2	1.6	1.4	1.2
Open-shell react. energies						
MAX	50.5	22.4	6.3	7.2	9.5	6.3
MUE	14.6	6.7	1.9	1.8	1.7	1.7
RMSD	18.0	8.9	2.5	2.5	2.7	2.4
Total						
MAX	53.7	23.7	8.8	9.9	10.2	8.4
MUE	17.1	7.4	2.2	2.4	1.9	1.7
RMSD	20.9	9.3	2.9	3.3	3.0	2.6

2 Structural determination

Table S5: Geometries of selected covalent molecules containing atoms of the second row of the periodic table from the SE100 database.[S9] Bond lengths in Å, valence and dihedral angles in degrees. All reference semi-experimental (SE) and rDSD/j3 geometries are taken from Ref. S9, except for CH_4 (SE Ref. S10, rDSD/j3 S8), H_2O_2 (SE Ref . S11, rDSD/j3 S8) and HNO (SE Ref. S12, rDSD/j3 S8).

		SE	B3LYP/j3	M062X/j3	rDSD/j3	PCS	CCSD(T)/j3	CCSD(T)/j3+CV2	junChS
CH_4	r(C-H)	1.0859	1.0884	1.0871	1.0894	1.0879	1.0894	1.0880	1.0856
CO_2	r(C-O)	1.1600	1.1605	1.1553	1.1651	1.1610	1.1669	1.1650	1.1588
HCN	r(C-H)	1.0651	1.0659	1.0666	1.0674	1.0661	1.0679	1.0668	1.0652
	r(C-N)	1.1533	1.1463	1.1425	1.1575	1.1531	1.1604	1.1579	1.1520
HNC	r(N-H)	0.9954	0.9969	0.9980	0.9973	0.9965	0.9975	0.9966	0.9949
	r(N-C)	1.1685	1.1645	1.1617	1.1717	1.1674	1.1758	1.1734	1.1679
H_2O	r(O-H)	0.9573	0.9618	0.9593	0.9610	0.9597	0.9611	0.9602	0.9563
	θ(H-O-H)	104.53	105.11	105.30	104.46	104.65	104.12	104.23	104.49
NH_3	r(N-H)	1.0110	1.0132	1.0123	1.0137	1.0121	1.0143	1.0131	1.0100
	θ(H-N-H)	106.94	107.19	107.08	106.70	107.01	106.34	106.51	106.82
C_2H_2	r(C-H)	1.0617	1.0618	1.0631	1.0639	1.0627	1.06441	1.0633	1.0617
	r(C-C)	1.2030	1.1966	1.1942	1.2062	1.2017	1.2105	1.2079	1.2023
C_2H_4	r(C-C)	1.3311	1.3250	1.3221	1.3317	1.3274	1.3382	1.3354	1.3300
	r(C-H)	1.0807	1.0825	1.0821	1.0835	1.0824	1.08344	1.0821	1.0803
	θ(H-C-C)	121.42	121.73	121.59	121.50	121.50	121.45	121.46	121.45
	θ(H-C-H)	117.16	116.54	116.83	117.01	117.00	117.09	117.08	117.11
H_2CO	r(C-O)	1.2047	1.2003	1.1961	1.2072	1.2030	1.2115	1.2095	1.2032
	r(C-H)	1.1003	1.1059	1.1032	1.1041	1.1031	1.1029	1.1015	1.1000
	θ(H-C-O)	121.65	121.94	121.70	121.78	121.79	121.69	121.68	121.71
CH_2O_2 (t-HCCOH)	r(C-O1)	1.1976	1.1978	1.1933	1.2019	1.1980	1.2039	1.2020	1.1963
	r(C-H1)	1.0918	1.0960	1.0948	1.0952	1.0944	1.0943	1.0931	1.0920
	r(C-O2)	1.3411	1.3450	1.3377	1.3450	1.3410	1.3478	1.3455	1.3393
	r(O2-H2)	0.9660	0.9704	0.9683	0.9697	0.9681	0.9698	0.9690	0.9649
	θ(H1-C-O1)	125.14	125.15	125.02	125.11	125.08	125.09	125.05	125.05
	θ(O2-C-O1)	124.81	125.17	124.81	125.05	125.01	124.99	124.98	124.86
	θ(H2-O2-C)	106.81	107.83	107.79	106.93	107.13	106.51	106.61	106.80
CH_2NH	r(C-N)	1.2709	1.2639	1.2607	1.2721	1.2678	1.2785	1.2759	1.2702
	r(N-H3)	1.0192	1.0206	1.0199	1.0211	1.0197	1.0220	1.0208	1.0177
	r(C-H4)	1.0897	1.0936	1.0922	1.0932	1.0921	1.0924	1.0911	1.0894
	r(C-H5)	1.0861	1.0892	1.0884	1.0888	1.0878	1.0880	1.0867	1.0852
	θ(C-N-H3)	110.33	111.41	110.99	110.37	110.60	109.89	110.03	110.34
	θ(H4-C-N)	124.18	124.70	124.42	124.47	124.45	124.36	124.34	124.29
	θ(H5-C-N)	118.76	118.94	118.92	118.72	118.75	118.64	118.67	118.71
BH_3NH_3	r(B-N)	1.6453	1.6589	1.6501	1.6546	1.6477	1.6565	1.6516	1.6433
	r(B-H)	1.2058	1.2069	1.2058	1.2108	1.2088	1.2104	1.2082	1.2063
	r(N-H)	1.0101	1.0151	1.0146	1.0149	1.0136	1.0152	1.0143	1.0116
	θ(H-B-N)	105.00	104.98	104.90	104.86	104.97	104.79	104.88	104.94
	θ(H-N-B)	110.97	111.00	110.83	110.96	110.90	111.03	110.99	110.95

Table S5: Geometries of selected covalent molecules containing atoms of the second row of the periodic table from the SE100 database.[S9] Bond lengths in Å, valence and dihedral angles in degrees. All reference semi-experimental (SE) and rDSD/j3 geometries are taken from Ref. S9, except for CH_4 (SE Ref. S10, rDSD/j3 S8), H_2O_2 (SE Ref . S11, rDSD/j3 S8) and HNO (SE Ref. S12, rDSD/j3 S8).

		SE	B3LYP/j3	M062X/j3	rDSD/j3	PCS	CCSD(T)/j3	CCSD(T)/j3+CV2	junChS
C_2H_4O (Oxirane)	r(C-O)	1.4274	1.4296	1.4151	1.4316	1.4267	1.4350	1.4321	1.4253
	r(C-C)	1.4609	1.4631	1.4636	1.4636	1.4591	1.4683	1.4654	1.4601
	r(C-H)	1.0816	1.0844	1.0830	1.0848	1.0838	1.0844	1.0831	1.0814
	θ(C-O-C)	61.56	61.55	62.28	61.48	61.51	61.54	61.55	61.62
	θ(C-C-O)	59.22	59.22	58.86	59.26	59.24	59.23	59.23	59.19
	θ(H-C-O)	114.87	115.10	115.15	114.89	114.95	114.87	114.91	114.94
	θ(H-C-H)	116.25	115.64	116.08	116.05	115.98	116.21	116.19	116.14
C_2H_4NH (Aziridine)	r(C-C)	1.4772	1.4799	1.4779	1.4803	1.4759	1.4843	1.4815	1.4763
	r(C-N)	1.4708	1.4701	1.4594	1.4735	1.4676	1.4799	1.4761	1.4689
	r(N-H)	1.0124	1.0137	1.0126	1.0149	1.0132	1.0159	1.0147	1.0115
	r(C-H1)	1.0804	1.0830	1.0817	1.0836	1.0826	1.0833	1.0820	1.0804
	r(C-H2)	1.0797	1.0820	1.0806	1.0823	1.0813	1.0819	1.0806	1.0791
	θ(C-N-C)	60.29	60.44	60.84	60.3	60.37	60.20	60.24	60.33
	θ(C-C-N)	59.86	59.78	59.58	59.85	59.81	59.90	59.88	59.83
	θ(C-C-H1)	117.99	117.99	117.71	117.88	117.87	117.86	117.84	117.84
	θ(C-C-H2)	119.36	119.86	119.69	119.64	119.66	119.53	119.54	119.57
	θ(C-N-H)	109.27	110.46	110.52	109.47	109.74	108.88	109.08	109.39
	θ(N-C-H1)	118.43	118.63	118.53	117.88	118.39	118.23	118.26	118.29
	θ(N-C-H2)	114.20	114.72	114.76	114.45	114.53	114.35	114.42	114.46
cyc$-C_3H_6$ (Cyclopropane)	r(C-C)	1.5024	1.5043	1.4986	1.5061	1.5009	1.5107	1.5072	1.5015
	r(C-H)	1.0790	1.0808	1.0795	1.0817	1.0806	1.0816	1.0802	1.0785
	θ(H-C-H)	114.87	114.22	114.67	114.75	114.68	114.93	114.89	114.83
CH_2F_2	r(C-F)	1.3532	1.3631	1.3529	1.3584	1.3562	1.3583	1.3565	1.3522
	r(C-H)	1.0868	1.0898	1.0889	1.0899	1.0888	1.0895	1.0882	1.0866
	θ(F-C-F)	108.29	108.46	108.16	108.35	108.39	108.30	108.32	108.35
	θ(H-C-F)	108.74	108.68	108.79	108.71	108.73	108.74	108.75	108.76
	θ(H-C-H)	113.48	113.55	113.39	113.52	113.42	113.47	113.40	113.33
HOF	r(F-O)	1.4345	1.4300	1.3989	1.4303	1.4288	1.4398	1.4380	1.4329
	r(H-O)	0.9686	0.9712	0.9684	0.9697	0.9684	0.9698	0.9690	0.9651
	θ(H-O-F)	97.86	98.66	99.48	98.13	98.19	97.79	97.85	97.94
CH_2CHF	r(C1-F)	1.3424	1.3493	1.3404	1.3453	1.3430	1.3470	1.3451	1.3409
	r(C1-C2)	1.3213	1.3174	1.3145	1.3235	1.3192	1.3290	1.3262	1.3208
	r(C1-H4)	1.0792	1.0811	1.0810	1.0821	1.0810	1.0818	1.0805	1.0789
	r(C2-H5)	1.0785	1.0801	1.0799	1.0811	1.0801	1.0811	1.0798	1.0782
	r(C2-H6)	1.0772	1.0790	1.0783	1.0801	1.0790	1.0801	1.0788	1.0771
	θ(F-C1-H4)	112.10	111.69	111.96	112.07	112.08	112.22	112.23	112.23
	θ(F-C1-C2)	121.72	122.08	121.95	121.96	121.97	121.78	121.79	121.77
	θ(C1-C2-H5)	121.32	121.70	121.20	121.48	121.47	121.42	121.41	121.38
	θ(C1-C2-H6)	118.95	119.28	119.25	118.94	118.96	118.93	118.97	118.95
NH_2OH	r(N-O)	1.4409	1.4448	1.4239	1.4420	1.4363	1.4483	1.4452	1.4368
	r(O-H4)	0.9578	0.9620	0.9597	0.9615	0.9599	0.9617	0.9608	0.9578
	r(N-H3)	1.0138	1.0163	1.0152	1.0165	1.0150	1.0173	1.0161	1.0136
	θ(H4-O-N)	102.10	102.77	103.33	102.22	102.43	101.87	101.99	102.31

Table S5: Geometries of selected covalent molecules containing atoms of the second row of the periodic table from the SE100 database.[S9] Bond lengths in Å, valence and dihedral angles in degrees. All reference semi-experimental (SE) and rDSD/j3 geometries are taken from Ref. S9, except for CH_4 (SE Ref. S10, rDSD/j3 S8), H_2O_2 (SE Ref . S11, rDSD/j3 S8) and HNO (SE Ref. S12, rDSD/j3 S8).

		SE	B3LYP/j3	M062X/j3	rDSD/j3	PCS	CCSD(T)/j3	CCSD(T)/j3+CV2	junChS
	θ(O-N-H3)	103.62	104.05	104.55	103.78	104.03	103.48	103.63	103.87
	φ(H3-N-O-H4)	124.85	124.54	124.35	124.84	124.65	125.12	125.00	124.79
BH_2OH	r(O-B)	1.3498	1.3527	1.3502	1.3569	1.3504	1.3596	1.3565	1.3485
	r(O-H1)	0.9558	0.9619	0.9599	0.9615	0.9599	0.9617	0.9609	0.9567
	r(B-H2)	1.1957	1.1956	1.1937	1.1982	1.1963	1.1978	1.1957	1.1938
	r(B-H3)	1.1899	1.1905	1.1885	1.1926	1.1907	1.1922	1.1901	1.1882
	θ(B-O-H1)	112.90	114.15	114.11	112.95	113.27	112.46	112.58	112.96
	θ(H2-B-O)	119.80	120.60	120.44	120.49	120.51	120.44	120.45	120.42
	θ(H3-B-O)	117.24	116.78	116.96	116.75	116.81	116.72	116.75	116.82
H_2O_2	r(O-O)	1.4524	1.4509	1.4225	1.4509	1.4469	1.4593	1.4568	1.4482
	r(O-H)	0.9617	0.9667	0.9640	0.9659	0.9644	0.9660	0.9652	0.9611
	θ(H-O-O)	99.76	100.74	101.54	100.12	100.28	99.80	99.89	100.22
	φ(H-O-O-H)	113.60	113.47	111.77	113.13	113.22	113.38	113.49	112.08
HNO	r(H-N)	1.0536	1.0615	1.0561	1.0554	1.0543	1.0553	1.0541	1.0515
	r(N-O)	1.2087	1.1981	1.1897	1.2095	1.2053	1.2149	1.2127	1.2060
	r(H-N-O)	108.12	108.86	108.52	108.18	108.35	107.98	108.06	108.27

Table S6: Geometries of selected covalent molecules containing atoms of the third row of the periodic table from the SE100 database.[S9] Bond lengths in Å, valence and dihedral angles in degrees. All reference semi-experimental (SE) and rDSD/j3 geometries are taken from Ref. S9, except for H_2S (SE Ref. S13, rDSD/j3 S8).

		SE	B3LYP/j3	M062X/j3	rDSD/j3	PCS	CCSD(T)/j3	CCSD(T)/j3+CV2	junChS
SO_2	r(S-O)	1.4308	1.4379	1.4247	1.4421	1.4365	1.4421	1.4392	1.4299
	θ(S-O-S)	119.31	119.14	118.87	119.28	119.25	119.33	119.34	119.29
H_2S	r(S-H)	1.3356	1.3415	1.3359	1.3383	1.3372	1.3383	1.3362	1.3356
	θ(H-S-H)	92.11	92.52	92.34	92.49	92.31	92.30	92.23	92.21
PH_3	r(P-H)	1.4117	1.4178	1.4114	1.4154	1.4132	1.4159	1.4130	1.4116
	θ(H-P-H)	93.42	93.48	93.45	93.73	93.55	93.59	93.44	93.42
H_2CS	r(C-S)	1.6093	1.6065	1.5974	1.6111	1.6048	1.6192	1.6152	1.6091
	r(C-H)	1.0854	1.0876	1.0870	1.0884	1.0874	1.0878	1.0865	1.0848
	θ(H-C-S)	121.73	122.17	122.03	121.92	121.92	121.85	121.86	121.82
CH_2PH	r(H1-C)	1.0810	1.0817	1.0817	1.0834	1.0823	1.0835	1.0821	1.0804
	r(C-P)	1.6661	1.6631	1.6550	1.6685	1.6609	1.6777	1.6730	1.6657
	r(P-H2)	1.4195	1.4257	1.4185	1.4221	1.4201	1.4224	1.4197	1.4183
	r(C-H3)	1.0829	1.0834	1.0833	1.0852	1.0842	1.0850	1.0837	1.0821
	θ(C-P-H2)	97.36	97.89	97.42	97.55	97.51	97.26	97.20	97.30
	θ(P-C-H1)	124.58	125.13	124.80	124.70	124.74	124.58	124.61	124.57
	θ(P-C-H3)	119.17	119.28	119.42	119.20	119.14	119.32	119.29	119.22
C_2H_4S (Thiirane)	r(C-S)	1.8111	1.8244	1.8016	1.8174	1.8092	1.8245	1.8195	1.8105
	r(C-H)	1.0798	1.0814	1.0805	1.0826	1.0815	1.0823	1.0810	1.0795
	θ(C-S-C)	48.25	47.81	48.51	48.18	48.25	48.10	48.13	48.31
	θ(H-C-S)	114.98	115.09	115.29	115.02	115.09	114.96	114.99	114.99
	θ(H-C-H)	115.66	114.97	115.32	115.48	115.42	115.63	115.59	115.57
H_2S_2	r(S-S)	2.0513	2.0721	2.0518	2.0609	2.0517	2.0722	2.0669	2.0518
	r(S-H)	1.3403	1.3464	1.3399	1.3422	1.3411	1.3419	1.3398	1.3396
	θ(H-S-H)	98.13	98.66	98.29	98.24	98.25	97.97	97.95	98.14
	φ(H-S-S-H)	90.72	90.71	90.86	90.63	90.61	90.68	90.73	90.66
CH_2Cl_2	r(C-Cl)	1.7640	1.7818	1.7662	1.7710	1.7640	1.7749	1.7708	1.7637
	r(C-H)	1.0816	1.0821	1.0822	1.0843	1.0831	1.0842	1.0829	1.0814
	r(Cl-C-Cl)	112.18	113.00	112.40	112.40	112.36	112.41	112.42	112.22
	r(H-C-Cl)	108.23	107.99	108.20	108.20	108.23	108.20	108.22	108.23
	r(H-C-H)	111.77	111.97	111.68	111.70	111.60	111.68	111.60	111.75
CH_2ClF	r(C-Cl)	1.7641	1.7853	1.7686	1.7723	1.7653	1.7745	1.7703	1.7642
	r(C-F)	1.3594	1.3662	1.3572	1.3638	1.3618	1.3649	1.3631	1.3583
	r(C-H)	1.0840	1.0857	1.0853	1.0869	1.0857	1.0867	1.0854	1.0838
	r(F-C-Cl)	110.02	110.35	109.95	110.13	110.16	110.07	110.09	110.07
	r(H-C-Cl)	107.96	107.75	107.97	107.99	108.03	108.04	108.06	107.95
	r(H-C-H)	112.57	112.83	112.59	112.64	112.54	112.60	112.52	112.56
HClCO	r(C-Cl)	1.7628	1.7855	1.7634	1.7702	1.7632	1.7704	1.7658	1.7608
	r(C-O)	1.1820	1.1787	1.1758	1.1857	1.1817	1.1892	1.1874	1.1808
	r(C-H)	1.0918	1.0944	1.0941	1.0946	1.0938	1.0941	1.0929	1.0918
	r(H-C-O)	126.38	126.73	126.36	126.35	126.30	126.09	126.04	126.31
	r(Cl-C-O)	123.33	123.65	123.28	123.54	123.50	123.51	123.52	123.34
	r(H-C-Cl)	110.29	109.61	110.37	110.11	110.19	110.40	110.44	110.35

3 Harmonic frequencies

Table S7: Comparison of computed and reference harmonic vibrational frequencies of molecules containing atoms of the second row of the periodic table, in cm^{-1}. For degenerate modes, each harmonic frequency is reported and included in the error analysis statistics reported in the main text.

Molecule	Mode	rDSD/3F12	rDSD/j3	Expt.
H_2O [S14]	$1a_1$	3820.7	3824.0	3832.2
	$2a_1$	1645.3	1644.9	1648.5
	$3b_2$	3933.8	3938.9	3942.5
HCN [S15]	1σ	3446.7	3445.4	3442.3
	2σ	2117.4	2111.3	2129.1
	3π	736.4/736.4	730.5/730.5	727.0
CO_2 [S16]	$1\sigma_g$	1351.4	1347.0	1352.8
	$2\sigma_u$	2404.6	2399.3	2392.2
	$3\pi_u$	671.9/671.9	667.3/667.3	672.9
H_2CO [S17]	$1a_1$	2930.0	2935.7	2931.9
	$2a_1$	1785.8	1783.5	1777.0
	$3a_1$	1536.5	1538.2	1535.2
	$4b_1$	1197.2	1199.3	1187.6
	$5b_2$	2996.6	3001.7	3006.9
	$6b_2$	1269.7	1268.1	1272.3
C_2H_2 [S18–S20]	$1\sigma_g$	3512.4	3512.2	3501.5
	$2\sigma_g$	2015.4	2012.6	2013.3
	$3\sigma_u$	3419.3	3419.1	3417.6
	$4\pi_g$	627.6/627.6	619.2/619.2	621.5
	$5\pi_u$	760.7/760.7	758.1/758.1	746.8
C_2H_2O [S21]	$1a_1$	3198.2	3203.3	3202.2
	$2a_1$	2204.2	2200.9	2197.2
	$3a_1$	1416.3	1418.0	1415.2
	$4a_1$	1160.9	1159.0	1146.0
	$5b_1$	589.6	587.1	581.9
	$6b_1$	530.1	520.8	502.6
	$7b_2$	3302.9	3307.9	3308.2
	$8b_2$	992.7	990.5	996.0
	$9b_2$	438.9	435.9	433.6
NH_3 [S22]	$1a_1$	3483.7	3484.5	3483.1
	$2a_1$	1044.4	1055.3	1052.8
	$3e$	3615.9/3615.9	3618.5/3618.5	3616.0
	$4e$	1675.3/1675.3	1675.8/1675.8	1675.7
BH_3 [S23]	$1a'$	2570.0	2574.3	2575.2
	$2a''$	1163.6	1164.6	1158.4
	$3e'$	2699.9/2699.9	2705.6/2705.6	2709.4
	$4e'$	1220.8/1220.8	1223.7/1223.7	1221.0
C_2H_4 [S24]	$1a_g$	3159.1	3164.7	3163.0
	$2a_g$	1684.4	1685.7	1676.5
	$3a_g$	1379.1	1380.3	1371.6
	$4a_u$	1064.8	1064.8	1052.2

Table S7: Comparison of computed and reference harmonic vibrational frequencies of molecules containing atoms of the second row of the periodic table, in cm^{-1}. For degenerate modes, each harmonic frequency is reported and included in the error analysis statistics reported in the main text.

Molecule	Mode	rDSD/3F12	rDSD/j3	Expt.
	$5b_{1u}$	3142.6	3146.4	3146.8
	$6b_{1u}$	1479.0	1483.3	1479.2
	$7b_{2g}$	966.0	962.3	955.3
	$8b_{2u}$	3249.2	3254.5	3254.5
	$9b_{2u}$	829.1	829.3	826.9
	$10b_{3g}$	3222.0	3227.2	3228.5
	$11b_{3g}$	1247.2	1248.7	1246.8
	$12b_{3u}$	976.3	979.7	967.8
CF_2 [S25]	$1a_1$	1242.0	1243.6	1246.2
	$2a_1$	675.7	676.4	674.9
	$3b_2$	1128.4	1132.1	1139.2
CH_4 [S26]	$1a_1$	3038.0	3042.1	3035.9
	$2e$	1571.5/1571.5	1574.0/1574.0	1571.8
	$3t_2$	3157.5/3157.5/3157.5	3160.9/3160.9/3160.9	3158.3
	$4t_2$	1348.4/1348.4/1348.4	1350.4/1350.4/1350.4	1343.1
CO [S27]	1σ	2165.8	2159.3	2169.8
HF [S27]	1σ	4106.7	4123.2	4138.3
HNO [S28]	$1a'a$	2961.6	2969.8	2954.2
	$2a'$	1610.9	1606.1	1603.8
	$3a'$	1548.4	1544.1	1546.5
N_2 [S27]	$1\sigma_g$	2337.7	2330.8	2358.6
N_2O [S29]	1σ	2273.2	2267.9	2282.1
	2σ	1319.7	1315.3	1298.3
	3π	598.9/598.9	599.3/599.3	596.3
H_2 [S30]	$1\sigma_g$	4430.1	4447.7	4401.2
$OH^{\bullet}$ [S30]	1σ	3753.3	3757.1	3737.8

Table S8: Comparison of computed and reference harmonic vibrational frequencies of molecules containing atoms of the third row of the periodic table, in cm^{-1}. For degenerate modes, each harmonic frequency is reported and included in the error analysis statistics reported in the main text.

Molecule	Mode	rDSD/3F12	rDSD/j3	Expt.
PH_3 [b] [S31]	$1a_1$	2427.8	2431.9	2434.4
	$2a_1$	1018.9	1022.5	1015.0
	$3e$	2436.0/2435.9	2439.9/2439.9	2426.7
	$4e$	1149.8/1149.8	1152.3/1152.3	1155.5
CCl_2 [S32]	$1a_1$	738.1	735.6	733.4
	$2a_1$	344.1	341.7	336.9
	$3b_2$	781.8	776.1	772.2
ClCN [S33]	1σ	2231.7	2227.0	2249.1
	2σ	751.5	751.2	747.7
	3π	390.0/390.0	392.2/392.2	381.8
Cl_2 [S27]	$1\sigma_g$	561.7	565.5	559.7
ClF [S34,S35]	1σ	793.1	787.2	783.5
CS [S27]	1σ	1297.1	1292.4	1285.1
H_2CS [S36]	$1a_1$	3083.7	3088.4	3088.2
	$2a_1$	1500.0	1503.1	1495.9
	$3a_1$	1091.8	1089.5	1075.5
	$4b_1$	1019.0	1023.6	1002.9
	$5b_2$	3172.5	3177.3	3180.7
	$6b_2$	1007.8	1008.6	1004.9
HCl [S27]	1σ	2998.8	3006.5	2991.0
HOCl [S37]	$1a'$	3784.6	3788.4	3797.1
	$2a'$	1272.6	1271.3	1272.1
	$3a'$	753.9	752.4	739.3
SiO [S35]	1σ	1224.9	1219.6	1241.5

References

(S1) Knizia, G.; Adler, T. B.; Werner, H.-J. Simplified CCSD(T)-F12 methods: Theory and benchmarks. *J. Chem. Phys.* **2009**, *130*, 054104.

(S2) Kállay, M.; Horvath, R. A.; Gyevi-Nagy, L.; Nagy, P. R. Size-Consistent Explicitly Correlated Triple Excitation Correction. *J. Chem. Phys.* **2021**, *155*, 034107.

(S3) Frisch, M. J.; Trucks, W., G.; Schlegel, B., H.; Scuseria, E., G.; Robb, A., M.; Cheeseman, R., J.; Scalmani, G.; Barone, V.; Petersson, A., G.; Nakatsuji, H.; Li, X.; Caricato, M.; Marenich, A. V.; Bloino, J.; Janesko, B. G.; Gomperts, R.; Mennucci, B.; Hratchian, H. P.; Ortiz, J. V.; Izmaylov, A. F.; Sonnenberg, J. L.; Williams-Young, D.; Ding, F.; Lipparini, F.; Egidi, F.; Goings, J.; Peng, B.; Petrone, A.; Henderson, T.; Ranasinghe, D.; Zakrzewski, V. G.; Gao, J.; Rega, N.; Zheng, G.; Liang, W.; Hada, M.; Ehara, M.; Toyota, K.; Fukuda, R.; Hasegawa, J.; Ishida, M.; Nakajima, T.; Honda, Y.; Kitao, O.; Nakai, H.; Vreven, T.; Throssell, K.; Montgomery, J. A., Jr.; Peralta, J. E.; Ogliaro, F.; Bearpark, M. J.; Heyd, J. J.; Brothers, E. N.; Kudin, K. N.; Staroverov, V. N.; Keith, T. A.; Kobayashi, R.; Normand, J.; Raghavachari, K.; Rendell, A. P.; Burant, J. C.; Iyengar, S. S.; Tomasi, J.; Cossi, M.; Millam, J. M.; Klene, M.; Adamo, C.; Cammi, R.; Ochterski, J. W.; Martin, R. L.; Morokuma, K.; Farkas, O.; Foresman, J. B.; Fox, D. J. Gaussian 16 Revision C.01. 2016; Gaussian Inc. Wallingford CT.

(S4) Kállay, M.; Nagy, P. R.; ; Rolik, Z.; Mester, D.; Samu, G.; Csontos, J.; Csónka, J.; Szabó, B. P.; Gyevi-Nagy, L.; Ladjánszki, I.; Szegedy, L.; Ladóczki, B.; Petrov, K.; Farkas, M.; Mezei, P. D.; ; Hégely, B. MRCC, a Quantum Chemical Program Suite. 2018.

(S5) Kállay, M.; Nagy, P. R.; Mester, D.; Rolik, Z.; Samu, G.; Csontos, J.; Csónka, J.; Szabó, B. P.; Gyevi-Nagy, L.; Hégely, B.; ; Ladjánszki, I.; Szegedi, L.; Ladóczki, B.;

Petrov, K.; Farkas, M.; Mezei, P. D.; Ganyecz, A. The MRCC Program System: Accurate Quantum Chemistry From Water to Proteins. *J. Chem. Phys.* **2020**, *152*, 074107.

(S6) Werner, H.-J.; Knowles, P. J.; Knizia, G.; Manby, F. R.; Schütz, M. Molpro: a General-Purpose Quantum Chemistry Program Package. *WIREs Comp. Mol. Sci.* **2012**, *2*, 242–253.

(S7) Werner, H.-J.; Knowles, P. J.; Manby, F. R.; Black, J. A.; Doll, K.; Heßelmann, A.; Kats, D.; Köhn, A.; Korona, T.; Kreplin, D. A.; Ma, Q.; Miller, T. F.; Mitrushchenkov, A.; Peterson, K. A.; Polyak, I.; Rauhut, G.; Sibaev, M. The Molpro Quantum Chemistry Package. *J. Chem. Phys.* **2020**, *152*, 144107.

(S8) Di Grande, S.; Kàllay, M.; Barone, V. Accurate Thermochemistry at Affordable Cost by Means of an Improved Version of the JunChS-F12 Model Chemistry. *J. Comp. Chem.* **2023**, *44*, 2149–2157.

(S9) Ceselin, G.; Barone, V.; Tasinato, N. Accurate Biomolecular Structures by the Nano-LEGO Approach: Pick the Bricks and Build Your Geometry. *J. Chem. Theory Comput.* **2021**, *17*, 7290–7311.

(S10) Pawłowski, F.; Jørgensen, P.; Olsen, J.; Hegelund, F.; Helgaker, T.; Gauss, J.; Bak, K. L.; Stanton, J. F. Molecular Equilibrium Structures From Experimental Rotational Constants and Calculated Vibration–Rotation Interaction Constants. *J. Chem. Phys.* **2002**, *116*, 6482–6496.

(S11) Baraban, J. H.; Changala, P. B.; Stanton, J. F. The Equilibrium Structure of Hydrogen Peroxide. *J. Mol. Struct.* **2018**, *343*, 92–95.

(S12) Demaison, J.; Csaszar, A. G.; Dehayem-Kamadjeu, A. The Case of the Weak N-X Bond: Ab Initio, Semi-Experimental, and Experimental Equilibrium Structures of XNO (X = H, F, Cl, OH) and FNO2. *J. Phys. Chem A* **2006**, *110*, 13609–13617.

(S13) Cook, R. L.; De Lucia, F. C.; Helminger, P. Molecular Force Field and Structure of Hydrogen Sulfide: Recent Microwave Results. *J. Mol. Struct.* **1975**, *28*, 237–246.

(S14) Strey, G. External Properties of Force constants. *J. Mol. Spectr.* **1967**, *24*, 87–99.

(S15) Strey, G.; Mills, I. M. The Anharmonic Force Field and Equilibrium Structure of HCN and HCP. *Mol. Phys.* **1973**, *26*, 129–130.

(S16) Gershikov, A. G.; Spiridonov, V. P. Anharmonic Force Field of CO_2 as Determined by a Gas-Phase Electron Diffraction Study. *J. Mol. Struct.* **1983**, *96*, 141–149.

(S17) Burleigh, D. C.; McCoy, A. B.; Sibert, E. L. An Accurate Quartic Force Field for Formaldehyde. *J. Chem. Phys.* **1996**, *104*, 480–487.

(S18) Temsamani, M. A.; Herman, M. The Vibrational Energy Levels in Acetylene $^{12}C2H_2$: Towards a Regular Pattern at Higher Energies. *J. Chem. Phys.* **1995**, *102*, 6371–6384.

(S19) Martin, J. M.; Lee, T. J.; Taylor, P. R. A Purely Ab Initio Spectroscopic Quality Quartic Force Field for Acetylene. *J. Chem. Phys.* **1998**, *108*, 676–691.

(S20) Strey, G.; Mills, I. M. Anharmonic Force Field of Acetylene. *J. Mol. Spectr.* **1976**, *59*, 103–115.

(S21) East, A. L.; Allen, W. D.; Klippenstein, S. J. The Anharmonic Force Field and Equilibrium Molecular Structure of Ketene. *J. Chem. Phys.* **1995**, *102*, 8506–8532.

(S22) Huang, X.; Schwenke, D. W.; Lee, T. J. Rovibrational Spectra of Ammonia. I. Unprecedented Accuracy of a Potential Energy Surface Used With Nonadiabatic Corrections. *J. Chem. Phys.* **2011**, *134*, 044320.

(S23) Schuurman, M. S.; Allen, W. D.; Schaefer III, H. F. The Ab Initio Limit Quartic Force Field of BH_3. *J. Comput. Chem.* **2005**, *26*, 1106–1112.

(S24) Martin, J. M.; Taylor, P. R. The Geometry, Vibrational Frequencies, and Total Atomization Energy of Ethylene. A Calibration Study. *Chem. Phys. Lett.* **1996**, *248*, 336–344.

(S25) Demaison, J.; Margules, L.; Martin, J.; Boggs, J. E. Anharmonic Force field, Structure, and Thermochemistry of CF_2 and CCl_2. *Phys. Chem. Chem. Phys.* **2002**, *4*, 3282–3288.

(S26) Wang, X.-G.; Carrington Jr, T. An Accurate Potential Energy Surface for Methane. 68th International Symposium on Molecular Spectroscopy. 2013; p EWG10.

(S27) Huber, K. Molecular Structure Constants of Diatomic Molecules. *Molecular Spectra and Molecular Structure Constants of Diatomic Molecules* **1979**,

(S28) Dateo, C. E.; Lee, T. J.; Schwenke, D. W. An Accurate Quartic Force Field and Vibrational Frequencies for HNO and DNO. *J. Chem. Phys.* **1994**, *101*, 5853–5859.

(S29) Teffo, J.-L.; Chedin, A. Internuclear Potential and Equilibrium Structure of the Nitrous Oxide Molecule From Rovibrational Data. *J. Mol. Spectr.* **1989**, *135*, 389–409.

(S30) Afeefy, H. Y.; Liebman, J. F.; ; Stein, S. E. In *NIST Chemistry WebBook, NIST Standard Reference Database Number 69*; Linstrom, P. J., Mallard, W. G., Eds.; National Institute of Standards and Technology, Gaithersburg MD, 20899, 2021; Chapter Neutral Thermochemical Data.

(S31) Wang, D.; Shi, Q.; Zhu, Q.-S. An *Ab Initio* Quartic Force Field of PH_3. *J. Chem. Phys.* **2000**, *112*, 9624–9631.

(S32) Tarczay, G.; Miller, T. A.; Czakó, G.; Császár, A. G. Accurate Ab Initio Determination of Spectroscopic and Thermochemical Properties of Mono-and Dichlorocarbenes. *Phys. Chem. Chem. Phys.* **2005**, *7*, 2881–2893.

(S33) Saouli, A.; Bredohl, H.; Dubois, I.; Fayt, A. FT Infrared Spectra of ClCN Between 1200 and 5000 cm^{-1} and Global Rovibrational Analysis of the Main Isotopomers. *J. Mol. Spectr.* **1995**, *174*, 20–50.

(S34) Bürger, H.; Jacob, E.; Fähnle, M. The Fourier Transform IR Spectra of ClF and BrF. *Zeitschr. Naturforsch. A* **1986**, *41*, 1015–1020.

(S35) Irikura, K. K. Experimental Vibrational Zero-Point Energies: Diatomic Molecules. *J. Phys. Chem. Ref. Data* **2007**, *36*, 389–397.

(S36) Yachmenev, A.; Yurchenko, S. N.; Ribeyre, T.; Thiel, W. High-Level Ab Initio Potential Energy Surfaces and Vibrational Energies of H_2CS. *J. Chem. Phys.* **2011**, *135*, 074302.

(S37) Skokov, S.; Peterson, K. A.; Bowman, J. M. An Accurate Ab initio HOCl Potential Energy Surface, Vibrational and Rotational Calculations, and Comparison With Experiment. *J. Chem. Phys.* **1998**, *109*, 2662–2671.
